# Single cell RNA sequencing of human microglia uncovers a subset associated with Alzheimer’s disease

**DOI:** 10.1038/s41467-020-19737-2

**Published:** 2020-11-30

**Authors:** Marta Olah, Vilas Menon, Naomi Habib, Mariko F. Taga, Yiyi Ma, Christina J. Yung, Maria Cimpean, Anthony Khairallah, Guillermo Coronas-Samano, Roman Sankowski, Dominic Grün, Alexandra A. Kroshilina, Danielle Dionne, Rani A. Sarkis, Garth R. Cosgrove, Jeffrey Helgager, Jeffrey A. Golden, Page B. Pennell, Marco Prinz, Jean Paul G. Vonsattel, Andrew F. Teich, Julie A. Schneider, David A. Bennett, Aviv Regev, Wassim Elyaman, Elizabeth M. Bradshaw, Philip L. De Jager

**Affiliations:** 1 Center for Translational and Computational Neuroimmunology, Columbia University Medical Center, New York, NY USA; 2 Taub Institute for Research on Alzheimer’s Disease and Aging Brain, Columbia University Medical Center, New York, NY USA; 3 Department of Neurology, Columbia University Medical Center, New York, NY USA; 4 Cell Circuits Program, Broad Institute, Cambridge, MA USA; 5 Edmond & Lily Safra Center for Brain Sciences, The Hebrew University of Jerusalem, Jerusalem, Israel; 6 Department of Pathology and Cell Biology, Columbia University Medical Center, New York, NY USA; 7 Institute of Neuropathology, Medical Faculty, University of Freiburg, Freiburg, Germany; 8 Berta-Ottenstein-Programme for Clinician Scientists, Faculty of Medicine, University of Freiburg, Freiburg, Germany; 9 Max-Planck-Institute of Immunobiology and Epigenetics, Freiburg, Germany; 10 Department of Neurology, Brigham and Women’s Hospital, Boston, MA USA; 11 Department of Neurosurgery, Brigham and Women’s Hospital, Boston, MA USA; 12 Department of Pathology, Brigham and Women’s Hospital, Boston, MA USA; 13 Signaling Research Centers BIOSS and CIBSS, University of Freiburg, Freiburg, Germany; 14 Center for NeuroModulation, Faculty of Medicine, University of Freiburg, Freiburg, Germany; 15 Rush Alzheimer’s Disease Center, Rush University Medical Center, Chicago, IL USA; 16 Klarman Cell Observatory, Broad Institute of MIT and Harvard, Cambridge, MA 02142 USA; 17 Howard Hughes Medical Institute, Department of Biology, MIT, Cambridge, MA 02140 USA; 18 Present Address: Genentech, 1 DNA Way, South San Francisco, CA 94080 USA

**Keywords:** Alzheimer's disease, Microglia, Neuroimmunology

## Abstract

The extent of microglial heterogeneity in humans remains a central yet poorly explored question in light of the development of therapies targeting this cell type. Here, we investigate the population structure of live microglia purified from human cerebral cortex samples obtained at autopsy and during neurosurgical procedures. Using single cell RNA sequencing, we find that some subsets are enriched for disease-related genes and RNA signatures. We confirm the presence of four of these microglial subpopulations histologically and illustrate the utility of our data by characterizing further microglial cluster 7, enriched for genes depleted in the cortex of individuals with Alzheimer’s disease (AD). Histologically, these cluster 7 microglia are reduced in frequency in AD tissue, and we validate this observation in an independent set of single nucleus data. Thus, our live human microglia identify a range of subtypes, and we prioritize one of these as being altered in AD.

## Abstract (web summary)

Imbalance of microglial phenotypes in the aging brain might underlie their involvement in late onset neurodegenerative diseases. Here we report the population structure of microglia in the aged human brain and the reduction of a particular microglia subset in individuals with Alzheimer’s disease .

## Introduction

Our understanding of microglia has evolved rapidly with respect to their ontology, role in developmental and physiological plasticity, as well as involvement in pathophysiology^1,2^. Recent transcriptome-wide studies of bulk ex vivo human microglia have consistently suggested that microglia change with age and have transcriptomes that are enriched for disease-related genes^3–5^. Further, we observed that one microglial transcriptional program in the aging dorsolateral prefrontal cortex (DLPFC) contributes to the accumulation of tau pathology while two others may relate to β-amyloid pathology^6^, highlighting the potential of different subsets of microglia being involved in different neuropathologies. However, these analyses used cortical-level bulk data, and the need for greater resolution led us to characterize heterogeneity of human microglia at the single-cell level. Recently, studies of single nucleus RNA sequencing from human brain reported either no detectable population structure in microglial cells^7,8^ or a maximum of four transcriptionally distinct microglial subsets^9^. The limited power of these studies to explore microglial heterogeneity comes from undersampling of both microglia as a cell population as well as the quality of their transcriptome. Here, we decided to explore microglial heterogeneity in the aging and AD human brain by targeting living microglial cells.

Genetic studies have highlighted a prominent role for microglia in susceptibility to different neurodegenerative diseases, particularly Alzheimer’s disease (AD)^10–12^. The concurrent pathologic processes taking place in the aging and AD cortex are known to create a diversity of contexts to which microglial cells can potentially contribute and respond to, suggesting that there may be a variety of microglial states with divergent homeostatic or pathophysiological roles in the older brain. This putative diversity of states makes targeting microglia in neurodegenerative diseases challenging: we need to understand the unique role of each subset in the pathogenesis and progression of age related neurodegenerative diseases. Then, we can carefully map which microglial subset to modulate in which direction in order to restore tissue homeostasis in brain aging and AD.

To explore microglial heterogeneity, we captured individual transcriptomes from 16,242 cells. These cells were purified using our validated experimental pipeline^5^, and they come from (1) autopsy samples of the DLPFC of 14 participants in the Memory and Aging Project (MAP), a study of cognitive aging that includes prospective brain collection^13,14^ and (2) 3 temporal cortex samples from individuals undergoing surgical resection for intractable epilepsy. These samples represent the two major types of tissue that are commonly used to extract live human microglia. 99.1% of these isolated cells express microglial marker genes, and our data identified multiple different subsets, yielding a catalog of 9 human microglial subpopulations that are present in both sets of samples. We illustrate the utility of our population structure model and associated analyses by assessing for enrichment in disease-related genes in each microglial subset and by performing a validation study using a targeted quantitative histological approach that shows a reduction in the frequency of one of these microglial subpopulations in individuals with AD. We then replicate this observation by repurposing DLPFC single nucleus RNA sequencing data generated from an independent set of MAP subjects^9^. Thus, we propose a model of microglial population structure and highlight one subtype whose frequency is altered in AD.

## Results

### Nature and distribution of the single-cell RNA sequencing data

**Fig. 1:**
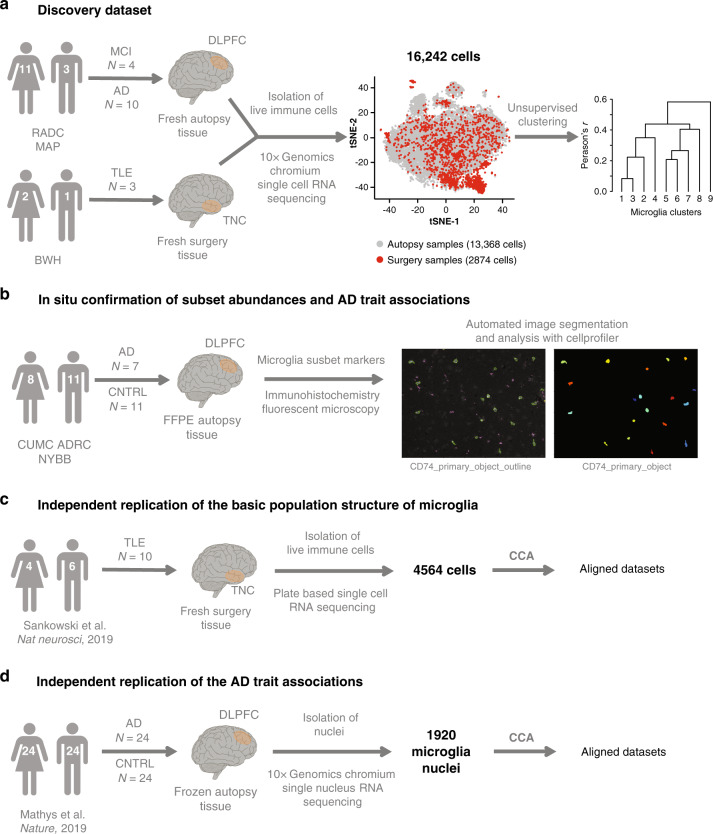
Experimental setup and overview of human samples and datasets used. **a** Workflow for the generation of the discovery dataset. Brain myeloid cells were isolated from 17 donors of both sexes (for a detailed isolation protocol see Methods section, for details on the donors see Supplementary Data 1). Autopsy samples originated from deceased aged individuals with various pathologies, while surgical biopsy samples were from young and middle-aged individuals undergoing surgery for intractable epilepsy. The single-cell suspension preparation of sorted cells was loaded onto one lane of the Chromium system (10x Genomics) and the resulting library was sequenced on the HiSeq4000 platform (Illumina). After quality control, the dataset consisted of 16,242 cells which were then subjected to unsupervised hierarchical clustering. **b** In situ confirmation of subset abundance and AD trait associations. We performed immunohistochemistry using markers enriched in microglial subsets in order to investigate the abundance of the specific clusters in situ and their associations to clinical and pathological traits of AD. Following image acquisition with a fluorescence microscope, automated image analysis was done using CellProfiler. **c** Independent replication of the basic population structure of microglia. We used a recently published human microglia single-cell RNA sequencing dataset to confirm the basic population structure of aged human microglia^25^. The two datasets were aligned using CCA. **d** Independent replication of the AD trait associations. A recently published single nucleus RNA sequencing dataset^9^ was used to confirm the AD trait associations found in our dataset. The two datasets were aligned using CCA. DLPFC dorsolateral prefrontal cortex, TNC temporal neocortex, MCI mild cognitive impairment, AD Alzheimer’s disease, TLE temporal lobe epilepsy, CNTRL non-neurological control, tSNE t-distributed stochastic neighbor embedding, RADC Rush Alzheimer’s Disease Center, MAP Rush Memory and Aging Project, BWH Brigham and Women’s Hospital, CUMC ADRC Columbia University Medical Center Alzheimer’s Disease Research Center, FFPE formalin fixed paraffin embedded, CCA canonical correlation analysis.

The primary goal of this report is to derive a model of microglial population structure with which we can generate and test disease-related hypotheses. Figure 1 details the workflow and the design of this study, which included a discovery dataset of single-cell RNA sequencing (scRNA-seq) from microglia (Fig. 1a) based on which we define the basic population structure of microglia, in situ confirmation of our findings (Fig. 1b) and independent replication of our observation in two independent datasets (Fig. 1c, d).

To minimize possible sources of heterogeneity, we have analyzed DLPFC samples obtained at autopsy from participants of the MAP study: thus, all subjects are part of the same prospective study of cognitive aging and are autopsied by the same team of neuropathologists at one site. They undergo the same, detailed, structured neuropathologic evaluation for aging-related pathologies. Further, all samples were processed by the same laboratory member in the same way to extract live immune cells (Methods), as previously validated^5^. Supplementary Data 1 outlines the demographic, clinical and neuropathological characteristics of each of the 14 profiled participants.

In parallel, we also processed another type of sample commonly used to extract human microglia: cortex resected in the vicinity of an epileptogenic focus during surgery for treatment-refractory temporal lobe epilepsy. Here, we processed fresh surgical samples from three different subjects, using the same experimental pipeline (Supplementary Data 1 contains a summary of the characteristics of these subjects).

The extracted live immune cells from these autopsy and surgery samples primarily consist of myeloid cells (Supplementary Fig. 1a), and we observed the presence of rare non-myeloid immune cells in some of the samples (Supplementary Fig. 1b). These non-myeloid cells were collected to serve as a positive control for our experimental and computational pipeline’s ability to resolve different cell types (Supplementary Fig. 1; see Supplementary Data 1 for sorting gate used with each sample). The purified cell suspension from each processed sample was profiled using the droplet-based Chromium platform from 10x Genomics (Methods section).

**Fig. 2:**
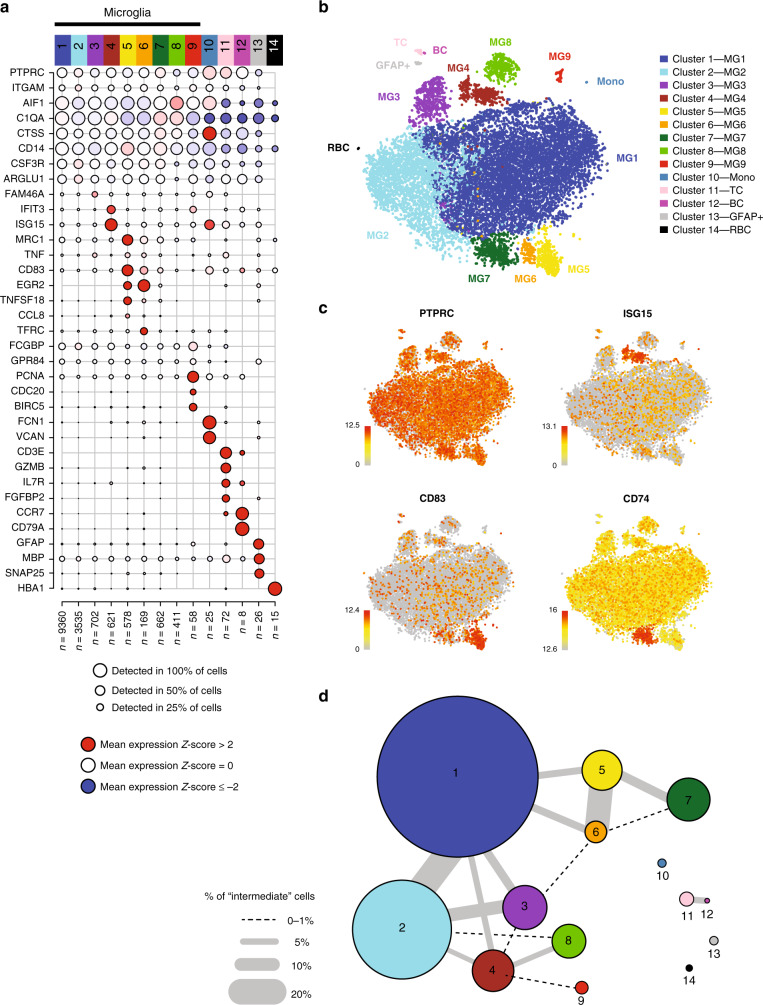
scRNA-seq identifies subsets of human brain myeloid cells. **a** Unsupervised iterative PCA-Louvain clustering with stepwise cluster robustness assessment identified 14 different clusters of cells in our dataset. Each column represents a cell cluster. The number of cells assigned to each cluster is noted at the bottom of each column. Each row represents the level of expression of a selected key gene. The size of the dot represents the fraction of cells in a given cluster in which the gene was detected (>0 transcripts per million). The color of the dot represents the average expression *z*-score (calculated over all 16,242 cells) of the cells within a given cluster. The bulk of the cells belong to 10 clusters (clusters 1–10) identifiable as myeloid based on their marker gene expression (*AIF1* and *CD14*). Of these, cluster 10 has low expression of *C1QA*, a microglia marker, and thus probably represents monocytes. A small proportion (<1%) of the cells are non-myeloid and belong to clusters that could be characterized by high expression of genes such as *GFAP* (cluster 13), *CD3E* (cluster 11), *CD79A* (cluster 12), and *HBA1* (cluster 14), likely representing astrocytes, T cells, B cells, and erythrocytes, respectively. The *z*-score matrix is available in Supplementary Data 16. **b** t-SNE plot depicting the different microglial and non-microglial cell subsets. Each dot represents a cell. The cells are color coded based on their cluster affiliation. t-SNE was run using all of the cells in the dataset. **c** t-SNE plots showing the expression of some of selected genes that are enriched in certain clusters. Each dot represents a cell. The normalized gene expression levels of the selected genes for each cell is projected onto the t-SNE plots. Color gradient bar represents log2(TPM + 1) which has been normalized, so that gray equals to 10th percentile expression value and red equals to maximum expressed value. **d** Constellation diagram showing the relatedness among clusters based on post-hoc classification of cells. For every pair of clusters, a bootstrapped random forest approach was run to classify each cell 100 times, using 75% of the cells as training data for each run. In the diagram, each node represents a cluster, scaled by the number of cells that belong to it, and each edge represents the fraction of cells that were ambiguously assigned i.e. assigned to the same cluster in fewer than 75 runs, for a given pair of clusters. The largest cluster (cluster 1) shares substantial ambiguously assigned cells with clusters 2 and 3, which may suggest a continuum of states among these three clusters. The other microglial clusters share fewer ambiguously assigned cells with cluster 1, while the monocyte and non-myeloid clusters all share no ambiguously assigned cells with any the microglial clusters. t-SNE t-distributed stochastic neighbor embedding, MG1-9 the nine microglial cell clusters, TC T cells, BC B cells, Mono monocytes, GFAP+a GFAP-positive ambiguous cluster; RBCs red blood cells.

A rigorous pre-processing pipeline (Methods section) yielded, from both sets of samples, transcriptomes from a total of 16,242 individual cells with a median of 833 cells sequenced per subject (Supplementary Data 2). The mean number of unique molecular identifiers (UMIs) and genes detected per cell in each subject (Supplementary Fig. 2a, b) was comparable within and between the two sets of donors. Each subject was processed for single-cell RNA-seq on a different day and is therefore its own batch. Accordingly, we performed robust batch correction on our data using a standard regression model (Methods section). Subsequently, we ran an iterative PCA-Louvain clustering approach^15,16^ with stepwise cluster robustness assessment and identified 14 distinct cell clusters with a minimum of 8 cells per cluster (Fig. 2a). Identifying the optimal number of cell clusters varies based on the termination criteria for clustering and the goal of the analysis. To facilitate the repurposing of our data set, we have made these single-cell data publicly available (see Data availability statement). Here, we elected to pursue a relatively conservative approach using a strict post-hoc machine learning method (Methods section) to assess cluster distinctness and clearly delineate the higher-order structure of human microglia. Nonetheless, in the future, larger sample sizes will allow us to develop a higher-resolution map that may reveal sub-cluster architecture.

In 10 out of the 14 clusters (99.25% of all cells), we detect known myeloid markers: *CD14* and *AIF1* (the gene encoding the protein IBA1; Fig. 2a). The remaining cells are distributed among a putative T cell cluster (cluster 11), a B cell cluster (cluster 12), and one minor ambiguous cluster (cluster 13) expressing myeloid markers (such as *AIF1, C1QA*) as well as high levels of *GFAP*, *MBP*, and *SNAP25* (Fig. 2a). The latter cluster could consist of cell doublets, but we cannot unambiguously call them as such based on numbers of genes or UMIs detected (Supplementary Fig. 2c, d). Additionally, we detect 15 cells (cluster 14) that are probably erythrocytes, based on hemoglobin expression. Among the 10 myeloid clusters, we found that cluster 10 expressed *C1QA*, a microglial marker, at very low levels, as well as high levels of monocyte-enriched genes such as *FCN1*, *VCAN*, and *LYZ* (Supplementary Fig. 3a), suggesting that this cluster may represent monocytes or monocyte-derived cells. By contrast, the remaining 9 clusters express high levels of microglia-enriched genes, such as *C1QA*, *C1QB*, *C1QC*, and *GPR34* (Supplementary Fig. 3b); we therefore deem these 9 clusters to be distinct clusters of microglial cells. Visualizing the cells in a t-SNE (t-distributed stochastic neighbor embedding) plot further supports the separation of the microglial and the non-microglial clusters (Fig. 2b) and the different microglia subsets from each other (Fig. 2c). Importantly, neither the modified gating strategy to include the peripheral immune cells nor the different cell sorters affected the basic population structure of microglia (Supplementary Fig. 1c–g).

We assessed inter-cluster relatedness using a post-hoc random forest-based machine learning approach to characterize how well individual cells could be unambiguously classified in each cluster (see Methods section)^17,18^. We visualized the results of this approach in a constellation diagram^18^ (Fig. 2d, Methods section), where the thickness of the line between two clusters is proportional to the number of cells that are ambiguously assigned (<75 times assigned to the same cluster out of 100 independent runs) between a pair of clusters, using 75% of the cells as a training set in each run. This constellation diagram shows that the non-microglial clusters (clusters 10, 11, 12, 13, and 14) are clearly distinct from the microglial clusters. By contrast, three of the largest microglial clusters (accounting for 83.7% of all cells and 84.5% of the microglia) – have a larger proportion of cells ambiguously classified among them. This inter-relatedness among clusters 1, 2, and 3 suggests that they comprise cells with closely related transcriptomic signatures. Likewise, clusters 5 and 6 appear to be strongly related to one another. The remaining microglial clusters show more distinct signatures. Interestingly, this assessment of cluster inter-relatedness does not support the concept of a single linear relationship among clusters; rather, it suggests that they may differentiate radially from a common cell fate into a number of distinct states.

**Fig. 3:**
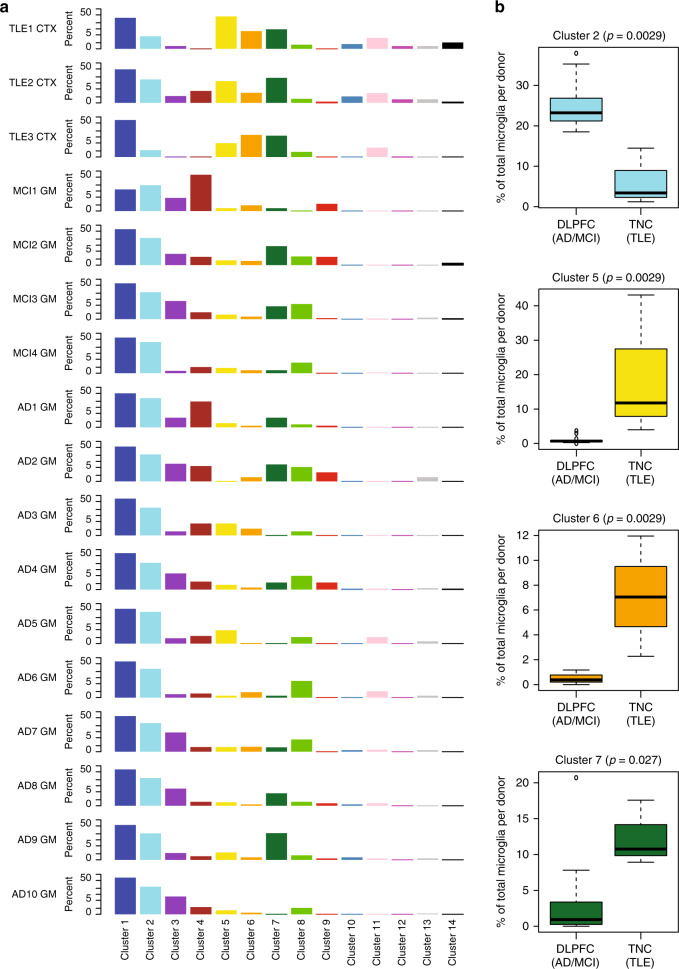
Cluster distribution within donors and provenance of clusters. **a** Distribution of cells among the different cell clusters for each donor. Each column represents a cluster, and each row represents a subject. The data are presented as the percentage of the cells in a given cluster within a given donor. Cluster 1 is the most abundant cluster in all subjects. Each cluster is color coded according to Fig. 2. **b** Clusters with differential proportions in the DLPFC (AD & MCI) autopsy samples versus the TNC (TLE) surgical tissue samples. Boxplots of the distribution of proportions of the 4 clusters with statistically significant differences between the two sample groups. The DLPFC (AD & MCI) group contains 14,142 cells from 14 donors (MCI1 GM - MCI4 GM, AD1 GM- AD10 GM), while the TNC (TLE) group contains 2103 cells from 3 donors (TLE1 CTX – TLE3 CTX). Significance was assessed using the (non-parametric) Mann–Whitney test, resulting in the *p*-values shown for each cluster. All tests were two-sided. The boxes represent the 25th percentile, median, and 75th percentile. The whiskers extend to the furthest value that is no more than 1.5 times the inter-quartile range (default parameter for R’s boxplot function). Source data for this figure are provided in the Source Data file. DLPFC dorsolateral prefrontal cortex, TNC temporal neocortex, MCI mild cognitive impairment, AD Alzheimer’s disease, TLE temporal lobe epilepsy, GM gray matter, CTX cortex.

We also assessed the extent of regional, intra- and inter-individual heterogeneity in the population structure of microglia in our data (Fig. 3 and Supplementary Figs. 4 and 5). We found that clusters 1 and 2 are the most abundant clusters in most individuals (Fig. 3a), and we therefore propose that clusters 1 and 2 may represent homeostatic microglial states, which fulfill routine, housekeeping tasks of the CNS parenchyma. Next, we found that there is inter-individual variability in the frequency of other microglial clusters (clusters 2, 5, 6, and 7; Fig. 3b). We note that there is some variability between the two sets of samples: the proportion of cells assigned to clusters 5 and 6 is greater in the surgical samples and cluster 2 is more frequent in the autopsy samples. Larger numbers of subjects and a greater sampling of tissue types and brain regions will be necessary to resolve whether these differences are due to age, sample type (autopsy *versus* surgery), brain regions (frontal versus temporal cortex), or the subject’s diagnosis. Nonetheless, all of the clusters are found in both sets of subjects (Fig. 3a and Supplementary Figs. 4 and 5).

To further address the issue of cluster robustness, we implemented a complementary strategy: we clustered selected samples in isolation, which also yielded significantly overlapping cluster signatures despite the much smaller number of cells available for analysis in each sample versus all samples (Supplementary Fig. 6). In this single-donor analysis (see Methods section), the median adjusted Rand index for the nine samples with the largest number of cells is 0.6790, suggesting that the clusters identified by this approach are robust within each subject: while some individuals have more cells in one cluster type, the clusters are not driven by single individuals. However, it is important to note the loss of power to detect some of the smaller clusters when analyzing samples separately, thus highlighting the value of analyzing all cells together when developing our population structure model. Overall, the proposed microglial cluster architecture appears to be present in all subjects.

### Annotating the clusters of human microglia

**Fig. 4:**
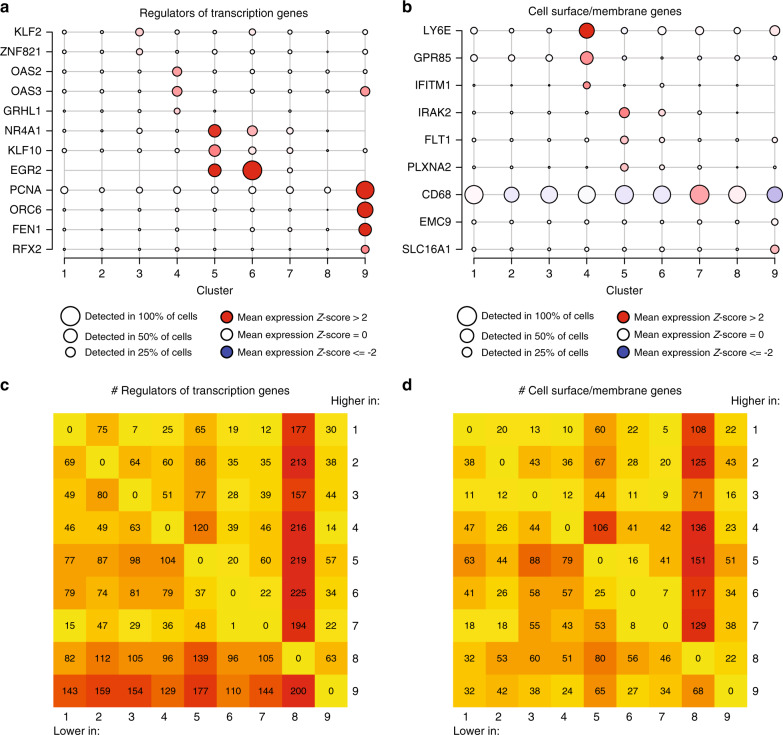
Identifying potential functional marker genes for the microglial clusters. **a** Microglial clusters are visualized in columns, and rows represent selected regulators of transcription that are differentially expressed in certain clusters (using the edgeR software package, adjusted *p*-value < 0.05, with Benjamini–Hochberg FDR correction). As shown in the key code at the bottom of the panel, the size of each dot represents the fraction of cells in a given cluster in which the gene was detected (>0 transcripts per million), and the color of the dot represents the mean of the expression *z*-score (calculated over all 16,242 cells) for the cells belonging to that cluster, as in Fig. 2a. **b** Using the same outline as in **a**, a subset of genes encoding membrane associated proteins that are differentially expressed across clusters are presented as these proteins are good candidates for cell-surface markers. The *z*-score matrix for **a** and **b** is available in Supplementary Data 16. **c**, **d** Heatmaps representing the number of differentially expressed genes in each pairwise comparison between the microglial clusters. In **c**, we limit the analysis to genes that encode transcription factors and transcriptional regulators. In **d**, we present the results of an analysis limited to genes encoding membrane associated proteins. Color scale for the heatmaps is yellow equals the minimum observed value (0), deep red equals the maximum observed value (225).

We next examined genes showing differential expression in the different microglial subtypes. Cluster-enriched sets of transcription factors and transcriptional regulators were found in some clusters (3, 4, 5, 6, and 9) but not in others (1, 2, 7, and 8; Fig. 4a). A similar pattern was observed for cell-surface markers (Fig. 4b). The lack of detectable distinct on-off transcription factors and cell-surface markers among clusters 1 and 2 is consistent with our hypothesis that these clusters may represent homeostatic microglia from which the other clusters differ by the upregulation of specific genes. From a global perspective (Fig. 4c, d), cluster 8 has the largest number of differentially expressed transcription factors and cell surface molecule-encoding genes (which were downregulated when compared to other clusters), whereas cluster 9 shows marked upregulation of transcriptional regulators. Cluster 3 was enriched in genes that have recently been shown to be induced by cellular stress in other human cells^19^ (Supplementary Fig. 7); thus this cluster could represent a subset of distressed cells. Supporting this claim, the abundance of cluster 3 was significantly reduced in the samples originating from surgery when compared to the autopsy samples (Supplementary Fig. 7g), and thus might be indicative of general cellular stress either due to aging or postmortem delay. Accordingly, we excluded this subset from subsequent functional annotation but report here the cluster and the gene set (Supplementary Data 3, 4, 5, and 6) that defines it as a specific reference signature for human microglial cells that are potentially altered in response to cellular stress.

**Fig. 5:**
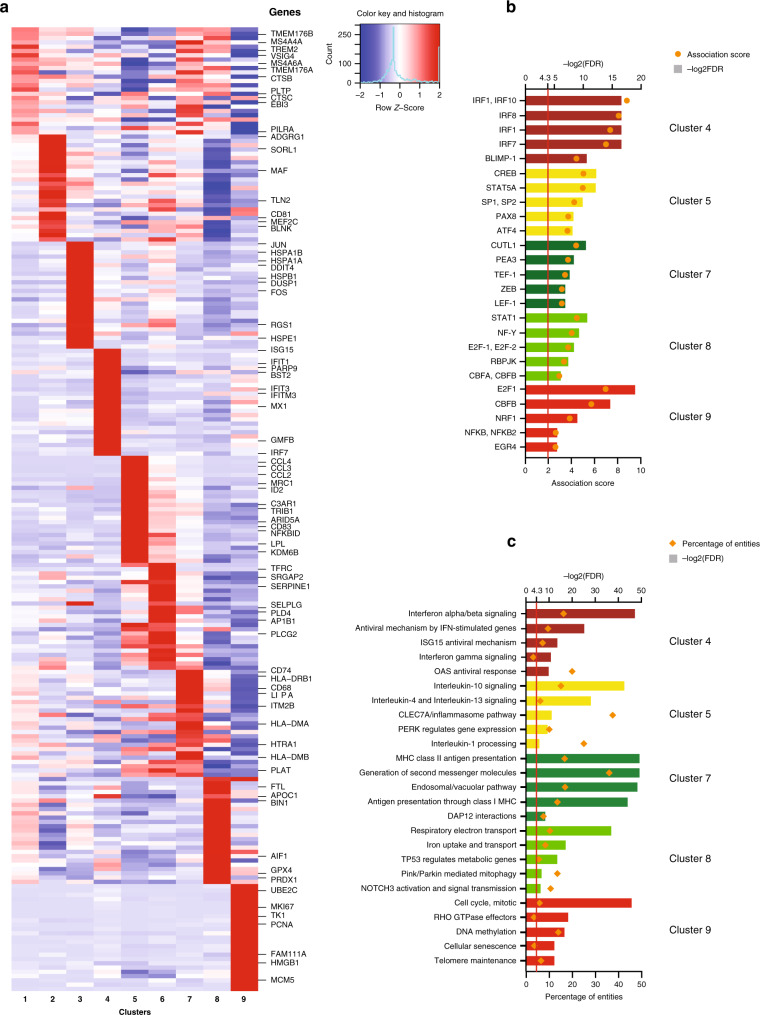
Functional annotation of the microglial clusters. **a** Heatmap depicting the *z*-scores of the top differentially expressed signature genes (using the edgeR software package, adjusted *p*-value < 0.05, with Benjamini–Hochberg FDR correction, ordered by adjusted *p*-value) of each microglial cluster, with representative genes highlighted on the right side of the figure. Rows represent genes, and each cluster is presented in a column. The color coding represents the mean expression (transcripts per million) in the cluster, *Z*-scored over all the clusters, as shown in the color key with histogram. The *z*-score matrix is available in Supplementary Data 16. **b** Predicted transcription factors whose binding sites are enriched among the differentially expressed genes of each microglial cluster. In rows, the names of the transcription factors are shown. Enrichment *p* values were calculated with PASTAA. The columns are colored according to the cluster identity introduced in Fig. 2. **c** Functional annotation of certain microglia clusters using REACTOME pathways significantly enriched for their signature genes (top 50 differentially expressed genes). The bar graphs are color coded according to cluster identity as introduced in Fig. 2. FDR false discovery rate.

For each of the remaining microglia clusters, we identified one set of cluster-defining genes (see Methods section and Supplementary Data 5) that were used in all subsequent analyses. Examples of genes belonging to these cluster-defining gene sets can be seen in Fig. 5a. First, we identified transcription factor binding sites which are enriched in the promoters of differentially expressed genes among these clusters. Using the PASTAA software package^20^, we observed some of the strongest enrichments for *CREB* and *ATF* transcription factors in cluster 5; *E2F1*, *CBFB* and *NRF1* in cluster 9; and *IRF* transcription factors (*IRF1, 7* and *IRF8*) in cluster 4 (Fig. 5b). The latter result is consistent with the excess of interferon response genes in this cluster (Fig. 5a and Supplementary Data 6). These results prioritize regulators that may play an important role in each of these microglial subsets. Gene set enrichment analysis^21^ (Fig. 5c and Supplementary Data 7) reveals that cluster 7 is enriched in genes related to antigen presentation, while the closely related clusters 5 and 6 feature genes related to anti-inflammatory responses (IL-10, IL-4, and IL-13). Cluster 4 is enriched in genes belonging to the interferon response signaling pathway, and cluster 9 is enriched in genes associated with the cell cycle, suggesting that it may constitute a pool of proliferating microglial cells.

**Fig. 6:**
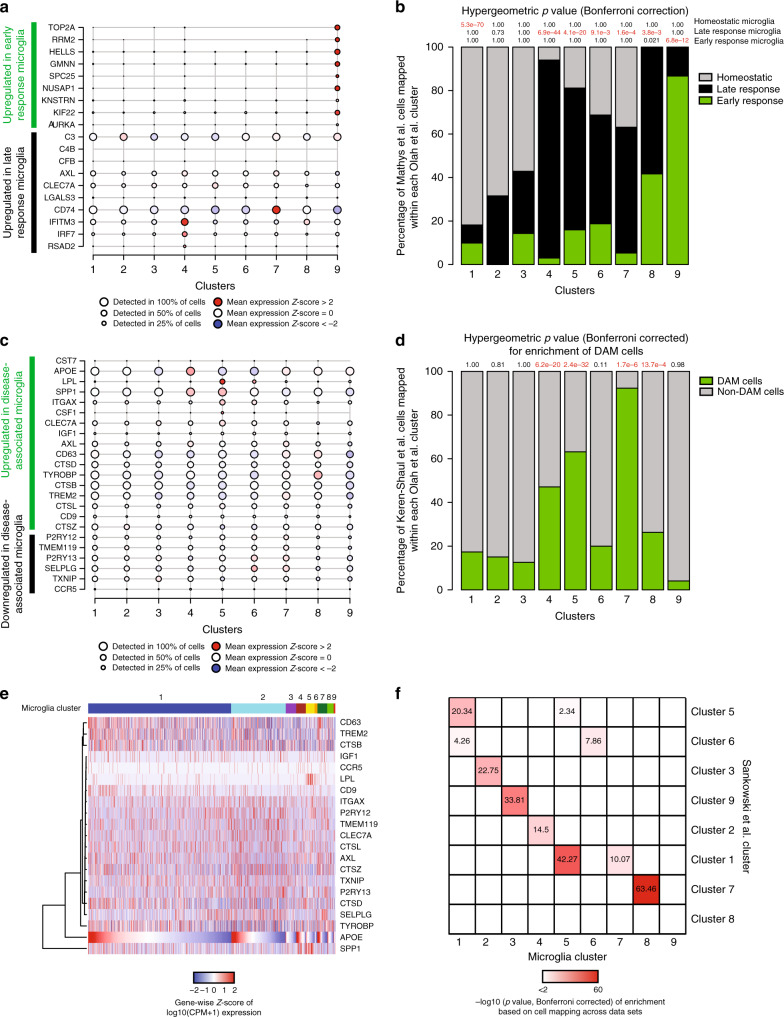
Annotation of the microglial clusters using published datasets. **a** Plot depicting the expression levels of representative genes upregulated in either the early (green) or late (black) response of microglia in the CKp25 mouse model^22^. Each cluster described in the current human study is presented in one column. The size of the dots is proportional to the number of cells expressing the given gene in the corresponding cluster. The color of the circle is proportional to the level of differential expression of the selected gene in a given microglial subset, with increased expression denoted in red while decreased expression is shown in blue. **b** We used Canonical Correlation Analysis (CCA)^37^ to map the mouse microglia to our single-cell microglia clusters using a Naïve Bayes classifier. The mouse microglia in the original paper were annotated as being either homeostatic (gray), or part of the late response (black) or part of the early response (green) based on their transcriptomic signature. Next, we assessed relative enrichment of each mouse microglial type in each of the human clusters using a hypergeometric test with Bonferroni correction, with significant results highlighted in red. The results are reported at the top of each column; for example, we see a significant (*p* = 5.3 × 10^−70^) excess of mouse homeostatic cells in human microglial cluster 1. **c** Plot depicting the expression levels of representative genes related to the murine DAM phenotype^24^ in each microglial cluster. Each cluster is presented in one column. Genes are either upregulated (green) or downregulated (black) in murine DAM cells. The size of the circles is proportional to the number of cells expressing the given gene in the corresponding cluster. The color of the dots represents the mean *Z* score of expression. The *z*-score matrix for **a** and **c** is available in Supplementary Data 16. **d** Results of dataset integration (using CCA) between the Keren-Shaul data^24^ and the current dataset: the percentage of DAM (green) or non-DAM (gray) cells assigned to each human cluster is shown. The results of the enrichment analysis (hypergegeometric test) are shown at the top. Significant results are highlighted in red. The human microglia clusters 4, 5, and 7 showed the strongest enrichment for the signature associated with the murine DAM phenotype. **e** Heatmap depicting the expression levels of the genes in the murine Disease Associated Microglia (DAM) gene set^24^. Each column represents a cell. Cells are ordered first based on cluster and then *APOE* expression within each cluster. The clusters are labeled at the top of the panel. Genes (rows) are ordered based on unsupervised hierarchical clustering (dendrogram on the left side of the graph). The color code represents *Z*-score of expression for each gene (i.e. normalized by row). While some of the DAM genes show some correlation in expression levels across cells, the gene set does not appear to be as coherent as it is in mice. The *z*-score matrix is available in Supplementary Data 16. **f** We compare our clustering results with those of an independent human microglia single-cell RNA-seq dataset^25^. CCA was used for this comparison, and each cell reports the results of an enrichment analysis for each human microglia clusters reported by Sankowski and colleagues in the microglial clusters that we have defined. The significant correlations are color coded based on the corresponding –log10 transformed *p*-value (hypergeometric test) of the overlap between the upregulated gene sets in each cluster. Overall, the independent dataset returned clusters, which are similar to the ones that we have defined. CPM counts per million.

We then turned to the annotation of our microglial clusters using other signatures reported in the literature. In a recent study of the CKp25 mouse model of primary neurodegeneration, a microglial subset enriched in interferon response genes was implicated in late microglial responses to this in vivo perturbation, and a different subset was implicated in the early response^22^. In our data (Fig. 6a, b), the mouse early response genes were only detected in our proliferative cluster 9, suggesting that the early microglial response in the CKp25 mouse model may involve a proliferative reaction. The late response signature appears more nuanced in humans, with component genes from the mouse study being found in either all human microglial clusters or limited to the interferon response-enriched cluster 4 (Fig. 6a, b and Supplementary Data 7). Similarly, a meta-analysis of all of the currently available major RNA-seq datasets of purified mouse microglia determined gene co-expression modules with unique functionality^23^. Here again, we find a co-expression module that captures the putative proliferating microglia of cluster 9 (Supplementary Fig. 8a) and an interferon response module (Supplementary Fig. 8b). The modules relating to LPS response (Supplementary Fig. 8c) or neurodegeneration in this analysis of murine data (Supplementary Fig. 8d) were not enriched in any of our human clusters.

We also specifically evaluated our human clusters in regards to a recent report of a disease-associated microglia (DAM) signature from mouse^24^ (Fig. 6c, d and Supplementary Fig. 9). Most of the DAM genes were detected in multiple microglial clusters (Fig. 6c, d and Supplementary Fig. 9a) and were expressed in all of the donors (Supplementary Fig. 9b). Correlations among DAM genes were weak throughout the data set (Fig. 6e). We found that cluster 7 showed the strongest enrichment for the DAM expression profile, with clusters 4, 5, and 8 also showing some degree of enrichment (Fig. 6c, d and Supplementary Data 8). Thus, while cluster 7 may be the most murine DAM-like cluster, the gene signature attributed to DAM in mice appears to be more distributed among the different subsets of human microglia.

### Assessing the generalizability of our microglial subsets

To evaluate the robustness and offer a measure of replication to our proposed human microglial population structure, we compared our clusters to those identified in another dataset: 15 samples from epilepsy and brain tumor resection from Freiburg using similar analytic methods on data obtained through a different cell isolation and cDNA library preparation protocol^25^.

In comparing our clusters to the clusters defined independently in the Freiburg samples, we find that, for 8 of our clusters (looking at columns in Fig. 6f), the cluster-specific upregulated genes are enriched in a single cluster defined independently in the Freiburg data set when the overlap is assessed using CCA and a hypergeometric test (Methods section, Supplementary Data 8). Only cluster 1 in our data appears to have been split between two clusters in the Freiburg data, and our cluster 9 does not appear to have a corresponding cluster in this dataset. Looking at the Freiburg clusters (rows in Fig. 6f), clusters Cluster1 and Cluster6 appear to be an amalgam of different clusters based on our data. Overall, given the smaller number of cells sampled by the Freiburg team, the different types of samples included in the two independent datasets, the different experimental pipelines, and the modest sample size of the Freiburg dataset, there is substantial similarity in cluster definitions, highlighting the robustness of our respective analyses. Thus, while investigators will certainly refine microglial subclusters in the future, our proposed higher-level architecture appears to have robust and replicable features across different tissue source (autopsy versus surgery) and different experimental and analytic approaches.

### In situ validation of human microglial subsets

**Fig. 7:**
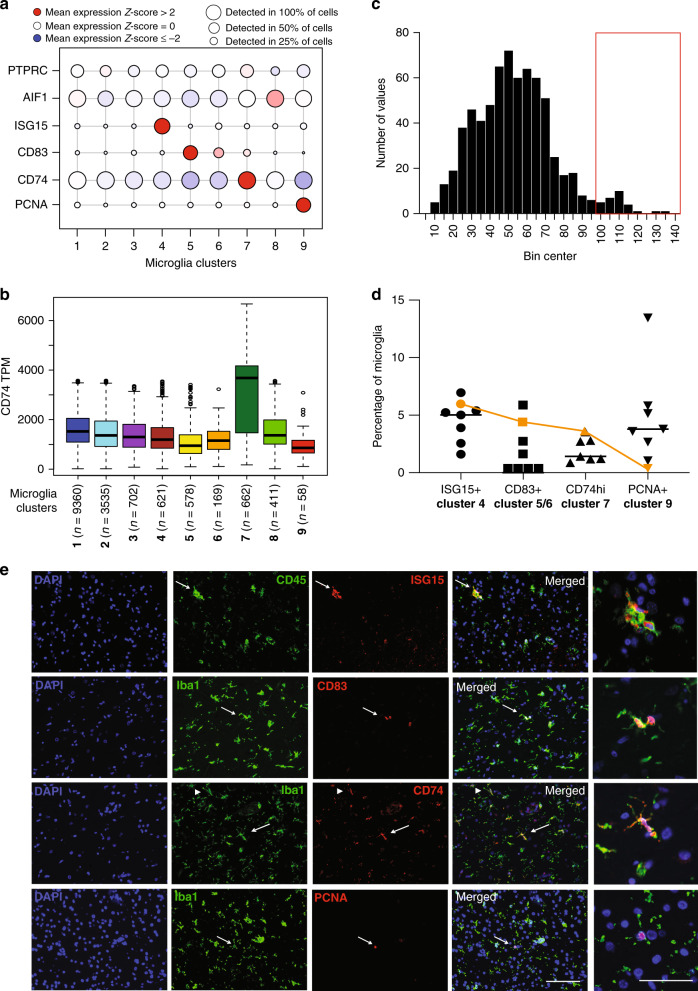
In situ confirmation of the abundance of major microglial subsets. **a** RNA expression levels of markers enriched in different subsets of microglia in the scRNA-seq dataset: *ISG15* for interferon cluster 4, *CD83* for the cytokine signaling enriched clusters 5 and 6, *CD74* for antigen presentation related cluster 7 and *PCNA* for the proliferative cluster 9. The size of the circles represent the percentage of cells per cluster in which the given gene was detected, while the color coding represent the normalized *z*-scores. The *z*-score matrix is available in Supplementary Data 16. **b** Box-and-whisker plot representing the normalized gene expression of *CD74* (in TPMs) among the different microglia clusters. Note that *CD74* gene expression is ~2-fold higher in cluster 7 when compared to the expression in other clusters (see Source Data file). The boxes represent the 25th percentile, median, and 75th percentile. The whiskers extend to the furthest value that is no more than 1.5 times the inter-quartile range (default parameter for R’s boxplot function). The number of cells in each microglial cluster is also shown. **c** Distribution of the expression levels of CD74 on microglia in situ as measured by immunofluorescence and CellProfiler analysis. Note the second small peak at high expression values that we highlight with a red box. Source data are provided in the Source Data file. **d** Black symbols represent the quantification of the ISG15+, CD83+, and PCNA+ and CD74^high^ microglia in the dorsolateral prefrontal cortex of seven individuals of mixed neuropathology (see Supplementary Data 9). The orange symbols represent the proportions for each subset observed in the single-cell RNA sequencing data. Center line represents the mean. Source data are available in the Source Data file. **e** Photomicrographs showing representative cells expressing the markers of the different microglia subsets. The arrows point to representative cells for each marker that are shown in the higher magnification photomicrographs in the far right column. In the micrographs showing CD74 staining, arrowhead points to a CD74 dim cell, while the arrow points to a CD74 bright (or high) cell. The bar in the lower right corner micrograph represents 100 μm for the overview images. The bar in the lower right corner of the higher magnification images (right most column) represents 50 μm. These experiments were performed in seven individual donors. In each donor 15–20 images were captured in the gray matter of the DLPFC and analyzed using IHC and automated image analysis. TPM transcripts per million.

To validate our microglial clusters in sections of frontal cortex from aging individuals (the same DLPFC region from which we extracted microglia from autopsy tissue), we selected genes that are proposed to mark different subsets of microglia; these include ISG15 for cluster 4, CD83 for clusters 5 and 6, CD74 for cluster 7 and PCNA for cluster 9 (Fig. 7a). Cluster 7 does not have a unique gene that is absent in all other clusters; rather, it is characterized by a marked upregulation (on average 2-fold greater in the scRNA sequencing dataset, Fig. 7b) of antigen processing and presentation related genes, such as CD74, when compared to the other microglial clusters. Thus, we elected to assign CD74^high^ cells - those microglial cells that have expression levels of CD74 protein greater than 2 standard deviations from the mean – as belonging to cluster 7 (Fig. 7c). Sections from the dorsolateral prefrontal cortex of individuals who either did (*n* = 4) or did not (*n* = 3) fulfill a pathologic diagnosis for Alzheimer’s disease (see Supplementary Data 9 for subject characteristics) were stained for the presence of ISG15, CD83, CD74, or PCNA and were co-labeled with anti-IBA1 or anti-CD45 antibodies (general markers of myeloid cells in the brain). Quantification of the double positive cells (for the general myeloid marker and for a marker enriched in one of the different microglia subsets) was performed using automated image segmentation and downstream quantitation by the CellProfiler software (https://cellprofiler.org/). We find that each of the four markers tags a subset of microglia. Specifically, an average of 4.38% (standard deviation (SD) = 1.83) of CD45^+^ cells express ISG15, a marker of the IFN response cluster 4 (Fig. 7d). In these subjects, we see substantial inter-individual heterogeneity in the frequency of cluster 4. This heterogeneity is somewhat less for the frequency of other clusters, with an average of 1.98% (SD = 2.46) of IBA1^+^ cells being CD83^+^ (marker for clusters 5/6, which have similar transcriptional signatures), and 4.60% (SD = 0.33) of microglia being IBA1^+^ CD74^high^ (antigen-presenting cluster 7)(Fig. 7d). In addition, we found 4.55% (SD = 4.00) of microglial cells to be IBA1^+^PCNA^+^ (proliferating cluster 9; Fig. 7d). Thus, for a subset of clusters with available markers (Fig. 5a), we confirm that the corresponding protein expression is restricted to a subset of microglia in the human brain (Fig. 7d, e), and we note that these cells have a morphology consistent with parenchymal ramified (CD83+, CD74high and PCNA+microglia cells) and ameboid (ISG15+) microglia (Fig. 7e). Since some clusters lack a clearly distinguishing signature (such as clusters 1, 2, and 8) in the future these clusters might be identified in situ by combinations of two genes or differing expression levels of multiple genes.

### Disease and trait associations of microglial subsets

**Fig. 8:**
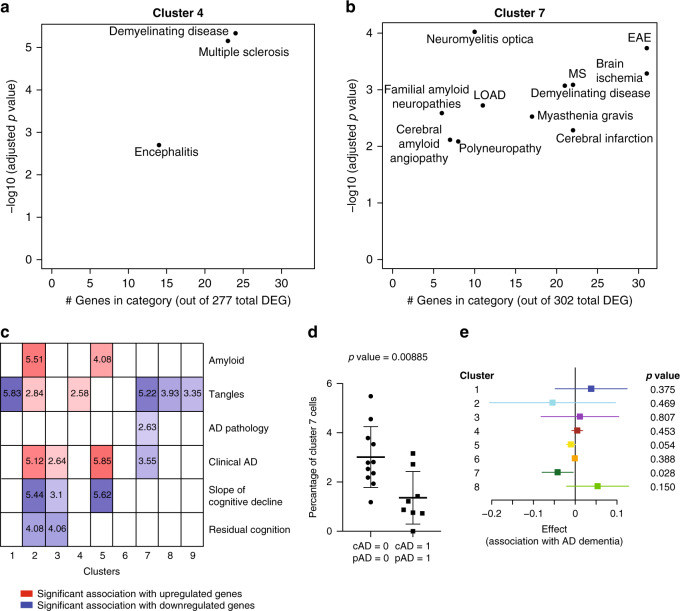
Disease association in human microglia clusters. **a**, **b** Scatter plots depicting brain related diseases – using gene sets from the disease ontology database (http://disease-ontology.org/) – that are significantly enriched (adjusted *p*-value < 0.01, hypergeometric test with Benjamini–Hochberg correction) in a given microglial cluster, using the cluster-defining signature gene sets of each microglia subset. Results for two different clusters are shown (cluster 4 and cluster 7); results for the other microglial clusters are included in Supplementary Fig. 10. In each plot, the *y*-axis reports the *p*-value of the enrichment analysis while the *x*-axis reports the number of genes that overlap between the cluster and disease gene sets, an indication of the robustness of the enrichment. **c** Panel reporting the result of enrichment analyses between the genes defining the microglial clusters and those genes that are associated with certain pathological or clinical traits found in the aging human brain (bulk DLPFC RNA sequencing data) in the ROS and MAP cohorts. Log10 adjusted *p*-values (using the hypergeometric test with Benjamini–Hochberg correction) are shown for those cluster/trait combinations where they are significant, and the saturation of each box is related to the strength of the association; red shades indicate overlap between cluster-defining genes and genes upregulated with the trait, whereas blue shades indicate overlap between cluster-defining genes and genes downregulated with the trait. **d** Dot plot comparing the frequency of IBA1+CD74^high^ cells within the IBA1+cells in DLPFC tissue sections from New York Brain Bank subjects with both AD dementia and a pathological diagnosis of AD (cAD = 1, pAD = 1; *n* = 8) to that found in subjects who fulfill neither of these diagnostic criteria (cAD = 0, pAD = 0; *n* = 11). Every dot is an individual donor (see Supplementary Data 9). Overlaid on the dot plot, data are also presented as mean values ± SD. The statistical test used was an unpaired *t* test with a two tailed *p* value. There is no difference in the frequency of IBA1^+^ cells (Supplementary Fig. 14a). See Supplementary Data 9 for demographics of the donors and Source Data file for raw data. **e** Forest plot presenting the effect size of the association statistic from an analysis comparing the frequency of a given microglial cluster in subjects with a diagnosis of AD dementia and a pathologic diagnosis of AD (cAD = 1, pAD = 1; *n* = 18) versus subjects that do not meet these diagnostic criteria (cAD = 0, pAD = 0; *n* = 20). The primary analysis involves cluster 7 to replicate results shown in panel **d**, and we also present results for the eight other microglial clusters that we have defined in this manuscript. The per individual proportions of each cluster is shown in Supplementary Fig. 14b. The mean of the coefficient (effect size) presented here is derived from a standard linear regression model (dependent variable = proportion of each microglial type over the total microglial nuclei for a donor, independent variable = AD pathology/dementia diagnosis, either 0 or 1, as in Fig. 8d). Bars in the forest plot represent the 95% confidence interval for the coefficient, and the *p*-value represents a two-sided *t*-test on whether the coefficient is significantly different from 0. *P*-values were Bonferroni corrected for multiple comparisons. Source data are provided as a Source Data file. DEG differentially expressed genes, AD Alzheimer’s disease, LOAD late onset Alzheimer’s disease, MS multiple sclerosis, EAE experimental autoimmune encephalomyelitis, cAD clinical diagnosis of AD dementia, pAD pathological diagnosis of AD.

Given that we have too few subjects to directly evaluate the association of microglial clusters to diseases and human traits in a robust fashion, we performed enrichment analyses (Methods section) to identify clusters that may be implicated in disease because they contain an excess of disease-associated genes. To assess statistical enrichment for disease-related genes, we used the DOSE Bioconductor package^26^, which contains curated lists of genes that are reported to be up- or downregulated in diseases, or positively or negatively associated with specific pathological traits. (Fig. 8a, b, Supplementary Fig. 10, and Supplementary Data 10). In this analysis, cluster 4 showed enrichment for multiple sclerosis, while 5 and 6 show enrichment for a large number of diagnoses, most prominently for neurovascular disease, encephalitis and neoplastic diseases but also multiple sclerosis and Alzheimer’s disease. Cluster 7 – the one most enriched for DAM genes – displays enrichment for inflammatory demyelination, ischemia and AD. On the other hand, peripheral myeloid cluster 10 also shows a mixed series of association with amyloid pathology, as well as inflammatory and proliferative diseases (Supplementary Fig. 11a). We also see that many individual GWAS-identified risk genes for AD, PD, MS, and ALS (Supplementary Fig. 12a through d, respectively) are expressed in most of the clusters and are not enriched in any particular microglial subset; however, microglial cells belonging to cluster 4 and 7 do have a higher expression of some of the AD susceptibility genes, such as *APOE* and *TREM2* respectively (Supplementary Fig. 12a). Further, cluster 4 has a higher expression of MS susceptibility genes (such as *IFITM3*; Supplementary Fig. 12c). Interestingly, the *TSPO* gene, the target for all current microglial markers used in positron emission tomography (PET) studies, is, at the RNA level, expressed in all clusters at a comparable level (Supplementary Fig. 12e) and may therefore be a good proxy for total microglial count.

We also explored the relationship between the different microglial clusters and aging-related traits (Fig. 8c and Supplementary Data 11). Since many of our samples came from autopsies of older individuals, we assessed for enrichment of gene signatures derived from our analyses of cortical tissue RNA-seq profiles in 541 aged individuals in each set of cluster-defining genes^27^, using a hypergeometric overlap approach taking into account the directionality of associations^28^. We evaluated genes associated with either a clinical or pathologic diagnosis of AD as well as with the molecularly specific measures of β-amyloid and PHF-tau tangle accumulation and the slope of cognitive decline before death^29,30^. We found that cluster 7 is the only cluster whose genes are altered in expression in the human cortex (DLPFC) in relation to both a pathologic diagnosis of AD and a diagnosis of AD dementia (Fig. 8c); specifically, the cluster 7 signature is reduced in expression in AD. In these samples, quantitative measures of the two component pathologies of AD (amyloid or tau) are also available, and a broader set of clusters are altered in relation to these pathologic measures, which are common in older adults without AD. Interestingly, we begin to see differences between the two pathologies, with clusters 2 and 5 being enriched for genes associated with β-amyloid while genes associated with PHF-tau are enriched in clusters 1, 2, 4, 7, 8, and 9. This suggests that different subsets of microglia may be involved in different aspects of AD. On the other hand, the monocyte cluster genes appear to be upregulated in relation to an increasing amyloid pathology burden (Supplementary Fig. 11d).

Finally, we also evaluated modules of co-expressed genes defined in the aging human frontal cortex^27^ that we had previously described as being enriched in microglial genes^6^. These five modules include m116, the cortical module most enriched for microglial genes, and m5, which is associated with both accumulation of tau pathology and the number of morphologically activated microglia in cortical tissue^6^. Gene-set enrichment analysis (Supplementary Fig. 12 and Supplementary Data 12) shows that m5 is widely expressed across microglial subtypes and does not align with any single microglial subset. This result suggests that morphologically activated microglia may exist in different transcriptomically defined states. This result is not surprising, as only those signatures shared by a large number of microglia will emerge in bulk tissue-level data. The important corollary to this point is that tissue-level data, while rich in many respects, is inadequate for the detailed investigation of the role of microglial subsets in neurodegenerative diseases and aging.

### Assessing the relation of IBA1^+^CD74^high^ cells to AD

Given that the set of genes that define the IBA1^+^CD74^high^ microglial cells of cluster 7 are enriched in AD-related genes (Fig. 8b) as well as the DAM signature (Fig. 6b) and that this gene set is downregulated in the cortex of individuals with a diagnosis of AD (Fig. 8c), we expanded our in situ study to include 8 cases of AD dementia that also fulfill a diagnosis of pathologic AD and 11 subjects that meet neither of these diagnostic criteria; all subjects underwent autopsy at the New York Brain Bank and were characterized in the same, structured manner (Supplementary Data 9). Adjusting for age and gender, we find that subjects with AD (1) have a significant reduction in the frequency of IBA1^+^CD74^high^ microglial cells in the frontal cortex (*p* = 0.0089; Fig. 8d) but (2) have no change in the number of total IBA1 + cells (*p* = 0.30; Supplementary Fig. 14a).

Finally, we repurposed a recently reported dataset of single nucleus data from frozen samples of the same brain region (DLPFC) of an independent set of MAP subjects^9^, and we used our cluster-defining gene sets to assign each microglia in the single nucleus dataset to one of our nine microglia clusters (see Methods section; Supplementary Fig. 14b and Supplementary Data 13). We find that, using our cluster-defining gene sets, these microglial nuclei are distributed to all of our clusters, except for the proliferative cluster 9. We then looked at the microglial nuclei assigned to cluster 7, and we performed an analysis comparing subjects with AD to control subjects which demonstrates that cluster 7 is reduced in frequency in these data in the context of AD (*p* = 0.028; Fig. 8e), confirming our histology-based analysis. Secondarily, we also evaluated the other clusters, but none are significantly altered in frequency in AD in this dataset.

We also explored in situ the distribution of cluster 7 cells as it relates to the topology generated by AD pathology in the aging brain. For this, we assessed the abundance of cluster 7 microglia within the perimeter of an amyloid plaque and outside of amyloid plaques using immunohistochemistry, fluorescence microscopy and automated image analysis using CellProfiler. These experiments were performed on DLPFC tissue sections from aged donors (for details see Supplementary Data 9). As shown in Supplementary Fig. 15d, we did not find any association between the topological distribution of cluster 7 and amyloid plaques. This is in line with our indirect analysis, which found no association between cluster 7 abundance and amyloid pathology (Fig. 8c).

## Discussion

This manuscript presents a new data set based on 16,096 individual microglial transcriptomes which, using unsupervised clustering, we segregate into 9 clusters of microglia. Our analysis identifies microglial subsets involved in homeostasis, proliferation, interferon response, and antigen presentation. Further, our enrichment analyses suggest that several of these clusters are enriched for genes involved in neurodegenerative diseases. Additionally, the clusters showed divergent associations to the pathological and clinical traits of AD. We have validated one of these observations histologically and with single nucleus data by demonstrating a reduced frequency of cluster 7 microglia in subjects with AD. To facilitate their use, our data are searchable at https://vmenon.shinyapps.io/microglia, and are available through the Synapse portal (https://www.synapse.org/#!Synapse:syn21438358). The counts matrices and the cell annotations can be found in Supplementary Data 14 and 15, respectively.

Prior studies have shown^31,32^ that the total number of human cortical microglia does not change in the context of AD or AD-related endophenotypes that are common in older individuals; rather, a small subset of morphologically activated microglia increased in frequency in relation to AD. Thus, unlike many mouse models with accelerated amyloid or tau proteinopathy, there does not seem to be a strong proliferative component to microglia in AD based on histological studies, and our single-cell data are consistent with these observations. While certain murine transcriptional programs – such as the DAM signature^24^ or the interferon response signature^22^ – are seen in our human microglia, they are distributed across different clusters, and the relevance of these models with accelerated protein aggregation over weeks to human AD which evolves over decades remains an open question in the AD field^33^.

By design, we have implemented a relatively conservative partitioning scheme to identify the higher-level architecture of human microglial subtypes from which further sub-clusters may be defined as sample sizes increase and other brain regions or diseases are investigated. This study serves as a framework with which to guide future study designs. Our report contains several insights: first, the nine different populations of microglia are found in both autopsy and surgical samples, and their frequencies are generally similar across both types of samples. Thus, these two major sources of primary, live microglia do not have large differences arising from technical factors or circumstances surrounding the agonal state for most microglial clusters; however, there may be an effect of smaller magnitude that will be detectable as larger datasets emerge. The clusters are also found in single nucleus data (Supplementary Fig. 14), consistent with the fact that our purification protocol does not appear to lose a specific microglial subtype.

Second, the different human microglial clusters show divergent enrichment for genes related to neurodegenerative disease and clinicopathological traits. Importantly, the signature of the murine Disease Associated Microglia (DAM) phenotype was present in several different human microglial subsets (clusters 4, 5, 7, and 8). Thus, the role of microglia in human disease is likely to be more nuanced than what has been described in the mouse to date. In particular, antigen-presenting cluster 7 stands out amongst the other clusters since it emerges as enriched for AD genes that are diminished in expression at the cortical tissue level in both pathologically defined AD and AD dementia (Fig. 8c and Supplementary Fig. 14b, c). This could happen if the subset of microglia corresponding to cluster 7 were diminished in frequency in the tissue in AD. This is what we found histologically: the frequency of cluster 7 microglia, as captured by the CD74^high^ marker, is reduced in tissue sections from individuals with both AD dementia and pathologic AD. This result is further validated in a repurposed single nucleus dataset of independent MAP participants. The latter data also show that other microglial clusters are not significantly altered in this set of samples, but other clusters such as cluster 8 that increases in frequency may become significant as sample sizes increase. This independent analysis of a recent single nucleus dataset^9^ illustrates how our model derived from living microglia can inform the analysis of a dataset limited by the quantity and quality of its single nucleus data: the lower quantity of RNA per cell and smaller number of sampled cells in the single nuclear data provided only a higher-order perspective in the published analysis. Our higher-resolution model enables us to more precisely map the microglial subset implicated in AD and to guide the selection of a key marker with which we can validate our observation histologically. It is likely that there may be additional microglial states to discover in other brain regions or other human samples, particularly in the context of development, infection, and neoplasia, which we have not sampled here.

Our study has certain limitations that result from the difficulty in obtaining the fresh autopsy samples and the use of a multi-step purification pipeline^5^ to isolate live microglia from human brain tissue. First, there may have been a survival bias among microglial subpopulations that are successfully profiled; this can be investigated further in the future using complementary approaches such as single nucleus RNA-sequencing. Our immunofluorescence studies begin to address this point and suggest that the frequencies of at least four of our clusters are consistent between our scRNA-seq and tissue imaging data. Second, our single-cell sample preparation process results in the loss of potentially important topological information from the tissue, and spatial transcriptomic approaches will be needed to more comprehensively localize putative microglial subtypes in tissue. Third, we have only sampled two cortical brain regions, and thus profiling a larger number of brain regions and subjects with different neurologic conditions is necessary to generate a more widespread reference of microglial states. Finally, most of the disease analyses reported here are indirect, relying on enrichment analyses, and they will need to be confirmed by direct analysis of cluster proportions in a large number of relevant samples.

Despite these limitations, our study opens several avenues of investigation: (1) the exploration of functions conducted by different microglial subtypes based on our transcriptomic analyses, (2) the generation of more complete transcriptomes, epigenomes, and proteomes to elucidate the function of each cluster now that we have markers with which to purify them (Fig. 2a), (3) enhanced in silico analyses of genetic, or tissue-level transcriptomic and epigenomic data to assess which microglial subtypes are involved in traits of interest, and (4) the identification of at least one subset of cortical microglia that may be related to AD and should be prioritized for further validation efforts. These avenues of investigation are crucial to developing a more comprehensive understanding of microglial diversity and function, which will drive the development of targeted microglial therapies, and this study thus provides an important step towards the overall goal of characterizing and manipulating microglia in human brain diseases.

## Methods

### Source of human brain specimens

The autopsy brain specimens originated from brain donation programs^34,35^ at Rush University Medical Center/Rush Alzheimer’s Disease Center (RADC) in Chicago, IL (Dr. Bennett) and at Columbia University Medical Center/New York Brain Bank in New York, NY (Drs. Vonsattel and Teich)^36^. The surgically resected brain tissue specimens originated from the Brigham and Women’s Hospital in Boston, MA from collaborators Drs. Sarkis, Cosgrove, Helgager, Golden, and Pennell. All brain specimens were obtained through informed consent and/or brain donation program at the respective organizations. All procedures and research protocols were approved by the corresponding ethical committees of our collaborator’s institutions as well as the Institutional Review Board (IRB) of Columbia University Medical Center (protocol AAAR4962). For a detailed description of the brain regions sampled, age of the donors, histopathology and clinical diagnosis please see Supplementary Data 1.

### The ROS and MAP cohorts at RADC

Some of the autopsy specimens used in this study (see Supplementary Data 1) originated from two prospective studies of aging: the Religious Orders Study (ROS)^35^ and the Memory and Aging Project (MAP)^34^. To enter these prospective studies, participants have to be at least 53 (ROS) or 55 (MAP) years old and non-demented at the time of enrollment. They are also required to sign an Anatomical Gift Act agreeing to donate their brain and spinal cord at the time of death. Each subject undergoes annual neuropsychologic evaluations while alive and a structured, quantitative neuropathologic examination at autopsy. A detailed description of the collected variables can be found at https://www.radc.rush.edu/docs/var/variables.htm. Brain specimens were distributed for this project from autopsies taking place Sunday morning to Thursday. Only autopsies for which the post mortem delay was less than 12 h were included in this study.

### Shipping of brain specimens

After weighing, the tissue was placed in ice-cold transportation medium (Hibernate-A medium (Gibco, A1247501) containing 1% B27 serum-free supplement (Gibco, 17504044) and 1% GlutaMax (Gibco, 35050061)) and shipped overnight at 4 °C with priority shipping.

### Microglia isolation and sorting

The isolation of microglia was performed according to our published protocol^5^, with minor modifications. In case of the cortical autopsy samples, the cortex (gray matter, GM) and the underlying white matter (subcortical white matter) were dissected under a stereomicroscope. The subcortical white matter samples were not used in this study. The epilepsy surgery samples of temporal lobe were processed without dissection as in this case the cortical white and gray matter was not always distinguishable. All procedures were performed on ice. The dissected tissue was placed in HBSS (Lonza, 10-508F) and weighed. Subsequently the tissue was homogenized in a 15-ml glass tissue grinder − 0.5 gm at a time. The resulting homogenate was filtered through a 70 um filter and spun down at 300 g for 10 min. The pellet was resuspended in 2 ml staining buffer (PBS (Lonza, 17-516 F) containing 1% FBS) per 0.5 gm of initial tissue and incubated with anti-myelin magnetic beads (Miltenyi, 130-096-733) for 15 min according to the manufacturer’s specification. The homogenate was than washed once with staining buffer and the myelin was depleted using Miltenyi large separation columns. The cell suspension was spun down and the cell suspension was then incubated with anti-CD11b AlexaFluor488 (BioLegend, 301318) and anti-CD45 AlexaFluor647 (BioLegend, 304018) antibodies as well as 7AAD (a dead cell marker, BD Pharmingen, 559925) for 20 min on ice. Subsequently the cell suspension was washed twice with staining buffer, filtered through a 70 um filter and the CD11b+/CD45+/7AAD- cells or CD45+/7AAD- cells (Fig. 1a, Supplementary Fig. 1, and Supplementary Data 1) were sorted on a BD FACS Aria II or Influx cell sorter. Cells were sorted in the A1 well of a 96 well PCR plate (Eppendorf, 951020401) containing 100 ul of PBS buffer and immediately submitted to single-cell capture, reverse transcription and library construction. The isolation protocol described above yields 50,000–500,000 live microglia per 0.5 g of cerebral cortical tissue, depending on the severity of neurodegenerative disease, tissue quality (affected by postmortem delay, storage) and handling of the tissue and cell suspensions during processing. All sorting was performed using 100 um nozzle. The sorting times varied according to the quality of the sample, but was on average between 10 and 20 min per sample. The sorting speed was kept between 8000 and 10,000 events per second.

### 10x Genomics chromium single-cell 3′ library construction

Viability was assessed by trypan blue exclusion assay, and cell density was adjusted to 175 cells per μl. In total, 7000 cells were then loaded onto a single channel of a 10x Chromium chip for each sample. The 10x Genomics Chromium technology enables 3′ digital gene expression profiling of thousands of cells from a single sample by separately indexing each cell’s transcriptome. First, thousands of cells are partitioned into nanoliter-scale Gel Bead-In-EMulsions (GEMs). Within one GEM all generated cDNA share a common 10x barcode. Libraries are generated and sequenced from the cDNA, and the 10x barcodes are used to associate individual reads back to the individual partitions. To achieve single-cell resolution, the cells are delivered at a limiting dilution. Upon dissolution of the Single Cell 3′ Gel Bead in a GEM, primers containing (i) an Illumina R1 sequence (read 1 sequencing primer), (ii) a 16 nucleotide 10x Barcode, (iii) a 10 nucleotide Unique Molecular Identifier (UMI), and (iv) a poly-dT primer sequence are released and mixed with cell lysate and Master Mix. Incubation of the GEMs then produces barcoded, full-length cDNA from poly-adenylated mRNA. After incubation, the GEMs are broken and the pooled fractions are recovered. Full-length, barcoded cDNA is then amplified by PCR to generate sufficient mass for library construction. Enzymatic fragmentation and size selection are used to optimize the cDNA amplicon size prior to library construction. R1 (read 1 primer sequence) are added to the molecules during GEM incubation. P5, P7, a sample index, and R2 (read 2 primer sequence) are added during library construction via end repair, A-tailing, adapter ligation, and PCR. The final libraries contain the P5 and P7 primers used in Illumina bridge amplification. The described protocol produces Illumina-ready sequencing libraries. A Single Cell 3’ Library comprises standard Illumina paired-end constructs which begin and end with P5 and P7. The Single Cell 3′ 16 bp 10x Barcode and 10 bp UMI are encoded in Read 1, while Read 2 is used to sequence the cDNA fragment. Sample index sequences are incorporated as the i7 index read. Read 1 and Read 2 are standard Illumina sequencing primer sites used in paired-end sequencing. Sequencing the library produces a standard Illumina BCL data output folder. The BCL data will include the paired-end Read 1 (containing the 16 bp 10x Barcode and 10 bp UMI) and Read 2 and the sample index in the i7 index read.

### Batch structure and sequencing

The fresh autopsy and surgical resection samples were processed on the day of receipt for microglia isolation, library construction, and sequencing. Accordingly, each sample constitutes one batch for all three procedures. All sequencing was performed on an Illumina HiSeq4000 machine. For specifics regarding the generated reads see Supplementary Data 2.

### Single-cell RNA-seq data processing and alignment

Barcoded reads were demultiplexed and aligned to the GRCh38 genome with Ensemble transcriptome annotation (downloaded June 2017, GRCh38.85) using CellRanger with default parameters. Only cells with >1000 UMIs and <10,000 UMIs were kept for clustering and downstream analysis.

### Batch correction

Batch identity and total UMI per cell were regressed out using the vars.to.regress option in the Seurat ScaleData function^16,37^. This approach regresses out gene expression variation due to batch identity and total UMIs detected in each cell. Because there is a confound between library preparation batch and sample in our study, this regression is likely to remove some degree of biological signal and perhaps reduce the observed diversity of gene expression across donors and samples. Despite this, we chose to perform this batch correction to eliminate any spurious clustering due to technical issues, keeping in mind that it may mask further heterogeneity in the sampled cells. However, this provides a conservatively pre-processed dataset that enabled us to uncover robust microglial clusters that are found across samples: we find no residual clustering per subject, which was our goal since each subject is his/her own batch.

### Clustering using an iterative PCA-Louvain approach

Putative cell types were identified using an iterative clustering approach. After regressing out batch and total UMI number, all genes with variance greater than the mean were used to cluster cells with the PCA-Louvain clustering approach, as implemented in the Seurat R package^16,37,38^. Clustering was repeated using all combinations of principal components (running from 5 to 15) and resolution parameters (0.2, 0.4, 0.6, and 0.8). For each principal component/resolution parameter pair, the overall cluster robustness was assessed by training a random forest classifier^17^ on half of the cells and predicting the cluster membership of the remaining half. Any clusters showing a minimum prediction accuracy below 75% (over 20 iterations) were merged. Note that this is a much stricter criterion than standard 4- or 5-fold cross-validation, which use 75% or 80% of the samples as training data. The principal component/resolution parameter combination that yielded the largest number of robust clusters was then selected, and each resulting cluster from this combination was iteratively subclustered using the same procedure, until no further robust sub-clusters were found.

### Identification of a cluster-defining gene sets

After clustering, differentially expressed genes were identified over all pairs of clusters using the edgeR package^39^ on the un-normalized count values. For downstream analysis, for a given cluster, we ranked genes by the number of significant pairwise comparisons i.e. the number of times a gene was deemed significantly upregulated in the cluster of interest versus other clusters, with the important additional criterion that the gene was not found to be downregulated with respect to any other cluster. This approach allowed us to identify sets of genes that were higher in a given cluster with respect to other clusters, while simultaneously not being expressed at a lower level when compared to any other cluster. These cluster-defining lists of differentially expressed genes are provided in Supplementary Data 5.

### Constellation diagram

The constellation diagram in Fig. 2d, showing the relationship among different clusters, was generated using a cross-validation machine learning approach, similar to that used to assess cluster robustness. For each pair of clusters, cells were classified using 4-fold cross-validation using a random forest classifier^17^ (trained on 75% of the cells). This process was repeated 100 times, resulting in a membership score for each cell belonging to one or the other cluster in the pair. Cells that were not unambiguously classified (>75 times out of 100) to the same cluster were called intermediate cells. For the constellation diagram, the edges between any two clusters represent the percentage of total cells (from the pair of clusters) that were called intermediate, and the size of the nodes represents the total number of cells originally assigned to that cluster.

### Dot plot representations

For dot plot representations, normalized expression values (using the Transcripts Per Million (TPM) approach) for a given gene were *z*-scored over all the cells belonging to all the clusters visualized, and then per-cluster means of the *z*-scored values were calculated and plotted using the color scheme shown in each figure. Sizes of the circles represent the number of cells in the cluster in which the gene was detected (TPM > 0).

### Cluster enrichment by sample condition

For Fig. 3b, we assessed which microglial clusters showed enrichment in either of the two sample groups: dorsolateral prefrontal cortex (DLPFC) from deceased aged individuals with Alzheimer’s disease (AD) or Mild Cognitive Impairment (MCI), and temporal neocortex (TNC) from surgically resected tissue from younger temporal lobe epilepsy (TLE) patients. This comparison was performed as follows: (1) for each cluster, we calculated the proportion of cells that belonged to that cluster in each individual sample; (2) we ran a non-parametric Kruskal–Wallis test with Benjamini–Hochberg correction to identify which clusters showed differential proportions across the three regions. The adjusted *p*-values that showed significance (*p* < 0.05) are shown in Fig. 3b. Non-significant *p*-values are not indicated. It is important to note that the sample groups differ along several variables, so we cannot conclude unambiguously whether the differences in cell type proportion are due to brain region, disease condition, or subject age.

### Comparison to other RNA-seq data sets

As shown in Fig. 6, we compared our putative microglial clusters to microglial subtype signatures found in three other published data sets, as well as to a larger single-nucleus RNA-seq data set (Fig. 7). These four data sets were Keren-Shaul et al.^24^ (accessible under accession code GEO: GSE98969), Mathys et al.^22^ (accessible using the accession number GEO: GSE103334), Sankowski et al.^25^ (available at the Gene Expression Omnibus under accession code GSE135437), and Mathys et al.^9^ (available at Synapse (https://www.synapse.org/#!Synapse:syn18485175) under the doi 10.7303/syn18485175). For each of these data sets, we integrated the single cells/nuclei identified as microglia in that study with our data using Canonical Correlation Analysis^37^ (CCA). We then mapped these cells/nuclei to our single-cell microglia clusters in CCA space using a Naïve Bayes classifier. Next, we assessed relative enrichment of each cluster type within each of our clusters using a hypergeometric test with Bonferroni correction. For the single-nucleus RNA-seq data from Mathys et al.^9^, we assessed the difference in distribution of cluster proportions across in donors with AD pathology and AD-dementia (*n* = 18) and donors with neither AD pathology nor AD-dementia (*n* = 20) using a linear model corrected for age and sex.

### Comparison of single-donor clustering to full clustering

The same clustering method was applied to subsets of the data in order to assess the robustness and reproducibility of clustering. This included running the clustering on cells from each donor individually. The overlap of clusters was assessed qualitatively using heatmaps (see Supplemental Fig. 6). From a quantitative standpoint, we calculated the Adjusted Rand Index (a standard measure to assess the similarity of two partitionings of a given set) between the clusters derived from data subsets and the clusters derived from the full data set. In all cases, the Adjusted Rand Index was above 0.5, suggesting that clusters were identified robustly in subsets of the data.

### Functional annotation of the different microglia subsets

A list of the top 50 genes that were enriched in the given clusters (top 50 genes for each cluster that were found to be upregulated in the given cluster in the most number of pairwise comparisons (Supplementary Data 5)) was submitted to the analysis of upstream transcriptional regulation using PASTAA^20^ and pathway enrichment in REACTOME^20^. For both analyses, the output entities (transcription factor binding site matrices and pathways) with an FDR < 0.05 are reported in Supplementary Data 6 and 7, respectively. Representative transcription factors and pathways are shown in Fig. 5b, c.

### Disease ontology analysis

For disease ontology analysis, we used a list of all genes in each cluster that showed statistically significant upregulation in comparison to at least one microglial cluster, with the constraint that the gene did not show significant down-regulation with respect to any other microglial cluster (Supplementary Data 5). Using these cluster-specific gene lists, we performed disease ontology analyses using the DOSE R package^26^ with standard parameters and Benjamini–Hochberg procedure (Fig. 8a, b and Supplementary Figs. 10 and 11a–c). We note that we used this strategy after considering the option of using genes that are uniquely expressed in each cluster; we ultimately discarded this option because the number of such genes was too small to support robust enrichment analyses. This results from the fact that closely related groups of cells often can only be identified uniquely by a combination of genes (as opposed to individual genes), a finding that has been reported in multiple studies examining neuronal subtypes using single-cell RNA-seq.

### Association of clusters with ROSMAP traits

We assessed the association of cluster signatures to a set of five cognitive and pathological traits defined in the ROSMAP study (Fig. 8c and Supplementary Data 11), as follows. In these analyses, we used the cluster-defining gene list reported in Supplementary Data 5. First, for each ROSMAP trait, we extracted two sets of gene lists form the tissue level RNA sequencing data from the dorsolateral prefrontal cortex available on 541 individuals from the ROSMAP cohort^27^: those that showed positive association with the trait of interest, and those that showed negative association with the trait. Finally, we assessed the overlap of the upregulated genes for each cluster with the positively- and negatively associated genes for each trait using a standard hypergeometric test. The selected traits were described in detail elsewhere^5,6,27,34,35^. Additionally, a detailed description of the used variables (amyloid, tangles, global AD pathology burden, final consensus cognitive diagnosis, random slope of global cognition) can be found on the searchable website https://www.radc.rush.edu/docs/var/varIndex.htm.

### Mapping of single-nuclei data to single-cell clusters

A recent publication^9^ generated single-nucleus RNA-seq data from dorsolateral prefrontal cortex in 48 donors (comprising subsets of donors with and without diagnoses of Alzheimer’s disease) from the ROSMAP cohort (data available at Synapse (https://www.synapse.org/#!Synapse:syn18485175) under the doi 10.7303/syn18485175). We appended the single nuclei identified as microglia in that study to our clusters using Canonical Correlation Analysis (CCA)^37^, followed by mapping nuclei to our single-cell microglia clusters in CCA space using a Naïve Bayes classifier (Supplementary Fig. 14b). We then assessed the difference in distribution of cluster proportions in donors with AD pathology and AD-dementia (*n* = 18) and donors with neither AD pathology nor AD-dementia (*n* = 20) using a linear model corrected for age and sex (Fig. 8e).

### Association of clusters with ROSMAP modules

We assessed the enrichment of cluster signatures in previously reported microglial modules derived from bulk data from the ROSMAP study^6,27^. For each cell, we calculated a hypergeometric *p*-value based on the overlap of genes detected in that cell and genes belonging to a given module from Mostafavi et al^27^. For each cluster, we aggregated the *p*-values from all cells, and we used a Mann–Whitney test to assess whether the distribution of log *p*-values was significantly different from log(0.01). The Mann–Whitney *p*-values were then Bonferroni corrected to obtain the final association scores. As expected, the microglial clusters showed enrichment for a subset of modules known to be enriched for microglial signatures (Supplementary Fig. 13 and Supplementary Data 12), whereas the non-microglial clusters showed much weaker associations.

### In situ confirmation of microglia subset abundances

We used formalin fixed paraffin embedded tissue sections from the prefrontal cortex (BA9) of 19 donors from the New York Brain Bank (for donor specifics see Supplementary Data 9). Immunohistochemistry was performed as described below. 12 μm thick sections of human prefrontal cortex were de-paraffinized with Xylene for 20 min. The sections were put through an ethanol series (ethanol 100%, ethanol 100%, ethanol 70% – 1 min for each) and re-hydrated in water (for 1 min). Subsequently, the slides were washed 3 times with phosphate buffered saline (PBS). Antigen retrieval was achieved by putting slides in pH 6.0 citrate buffer and using microwave for 25 min at 400 Watt. The slides were placed in tap water for 5 min, washed three times with PBS. Unspecific binding of antibodies was blocked with 3% bovine serum albumin (BSA) in PBS containing 0.1% TritonX for 20 min. Primary antibody was applied overnight. Subsequently the slides were washed with PBS three times and the fluorochrome conjugated secondary antibody was applied to the slides for one hour. The slides were again washed three times with PBS. Endogenous autofluorescence was quenched with sudan black for 10 min. The slides were again washed with BPS three times and mounted with ProlongGold containing DAPI. For the amyloid staining, heat-induced epitope retrieval was performed using citrate pH = 6 using microwave oven (800 Watt, 30% power setting) for 25 min.

Then the sections were treated with formic acid for 2 min and blocked with blocking medium (3% BSA) for 30 min at room temperature (RT). The slides were incubated with primary antibodies overnight at 4 °C, followed with washes and incubation with secondary antibodies as described above.

The primary antibodies used were rabbit anti-human Iba1 (Wako; 019-19741; at the dilution of 1:500), red fluorochrome (635) conjugated anti-Iba1 (Wako; 013-26471; 1:500), mouse anti-human CD45 (Novus; NB500-319; 1:200), rabbit anti-human ISG15 (Proteintech; 15981-1-AP; 1:100), mouse anti-human CD83 (BioLegend; 305302; 1:100), mouse anti-human CD74 (BioLegend; 326802; 1:100), rabbit anti-human CD74 (Sigma; HPA010592; 1:100), mouse anti-human PCNA (Invitrogen; 13-3900; 1:100), and mouse anti-human beta-amyloid (Biolegend; 805501; 1:500). The secondary antibodies used were goat anti-mouse IgG (H+L) highly cross-adsorbed secondary antibody conjugated to Alexa Fluor Plus 488 (ThermoFisher Scientific; A32723; 1:300) or Alexa Fluor Plus 555 (ThermoFisher Scientific; A32727; 1:300) and goat anti-rabbit IgG (H+L) highly cross-adsorbed secondary antibody conjugated to Alexa Fluor Plus 488 (ThermoFisher Scientific; A32731; 1:300) or Alexa Fluor Plus 555 (ThermoFisher Scientific; A32732; 1:300). Nuclei were counterstained with DAPI (Invitrogen; P36931).

Photomicrographs were captured with a ×20 objective using Leica DMI 6000b fluorescence microscope. In all, 20 images were obtained from each donor from the gray matter of the prefrontal cortex. The images were then exported to ImageJ image analysis software (NIH, Maryland, USA) for further processing, before being loaded to the CellProfiler software where automated segmentation of cells and amyloid plaques was performed, followed by quantification of subset abundances as described in detailed below.

### Automated image analysis using CellProfiler

Immunofluorescence images were analyzed using the CellProfiler^40^ software to measure and classify cells according to the expression of the selected markers. First, the software was trained to automatically segment the images into: (1) cells (DAPI positive objects); (2) microglial cells (DAPI+/IBA1+ or DAPI+/CD45+ objects); (3) microglial subtypes (DAPI+/IBA1+/CD83+ and DAPI+/IBA1+/CD83−; DAPI+/IBA1+/PCNA+ and DAPI+/IBA1+/PCNA−; DAPI+/CD45+/ISG15+ and DAPI+/CD45+/ISG15−). The abundance of the microglia subsets was then expressed as percentage of ISG15 (cluster 4), CD83 (clusters 5/6), or PCNA (cluster 9) positive microglia over the total microglial population. Since CD74 was generally expressed by all microglia but was significantly upregulated in cluster 7 microglia, we elected to quantify the CD74^high^ microglia cells, which are defined as those microglial cells whose CD74 expression was higher than the mean CD74 expression plus two times the standard deviation. This cut off was in line with the distribution of the CD74 expression in microglia, as there was a second peak on the histogram that started emerging at these intensity values (see Fig. 7c). Thus for assessing the abundance of cluster 7 we quantified the percentage of DAPI+/IBA1+/CD74^high^ cells among the DAPI+/IBA1+cells. Similar approach was used for in situ confirmation of the abundance of cluster 4 microglia cells (ISG15+ subpopulation). To assess the abundance of the different microglia subsets in situ (Fig. 7d) formalin fixed paraffin embedded DLPFC tissue sections from 7 donors (see Supplementary Data 9). To investigate the differences in the abundance of cluster 7 microglia between AD and healthy control donors (Fig. 8d) we have assessed the abundance of CD74^high^ microglia (percentage of DAPI+/IBA1+/CD74^high^ cells among the DAPI+/IBA1+cells) in 19 donors (see Supplementary Data 9) with and without the clinical and pathological diagnosis of AD. Similar approach was used for assessing topological distribution of cluster 7 microglia (Supplementary Fig. 15) as it relates to amyloid plaques.

### Reporting summary

Further information on research design is available in the Nature Research Reporting Summary linked to this article.

## Supplementary information

Supplementary Information

Description of Additional Supplementary Files

Supplementary Data 1

Supplementary Data 2

Supplementary Data 3

Supplementary Data 4

Supplementary Data 5

Supplementary Data 6

Supplementary Data 7

Supplementary Data 8

Supplementary Data 9

Supplementary Data 10

Supplementary Data 11

Supplementary Data 12

Supplementary Data 13

Supplementary Data 14

Supplementary Data 15

Supplementary Data 16

Reporting Summary

## Data Availability

Our single-cell based transcriptomic data from human microglia is available in the form of a browsable platform at https://vmenon.shinyapps.io/microglia. The raw data files are available through Synapse (https://www.synapse.org/#!Synapse:syn21438358). The raw data of the Sankowski et al.^25^ study are available at the Gene Expression Omnibus under accession code GSE135437. The raw data from Mathys et al.^22^ are available using the accession number GSE103334.The raw data from Keren-Shaul et al.^24^ are accessible under accession code GEO: GSE98969. The Mathys et al.^9^ data are available at Synapse (https://www.synapse.org/#!Synapse:syn18485175) under the doi 10.7303/syn18485175. Source data are provided with this paper.

## Source data

Source Data

## Code availability

All analysis R code is available as a github repository (https://github.com/vilasmenon/Microglia_Olah_et_al_2020).
