# Supplementary material for: Single cell RNA sequencing of human microglia uncovers a subset associated with Alzheimer’s disease
Source: Nat Commun. 2020 Nov 30;11:6129. doi: 10.1038/s41467-020-19737-2 (PMC7704703; doi:10.1038/s41467-020-19737-2)
Supplement: Supplementary file 1 — Supplementary Information [file 41467_2020_19737_MOESM1_ESM.pdf]

Single cell RNA sequencing of human microglia uncovers a subset associated with Alzheimer's disease

Olah et al.

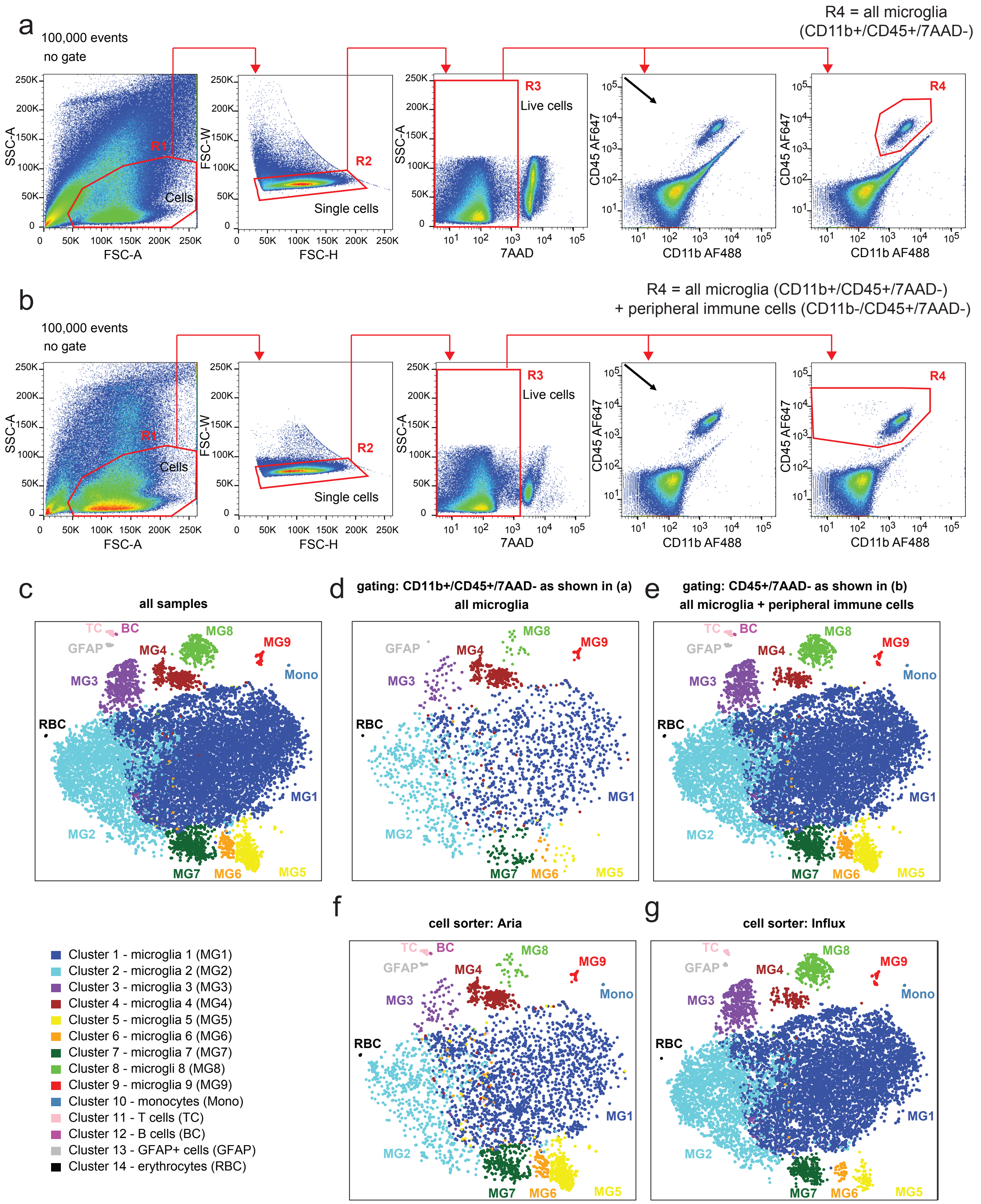

Supplementary figure 1. Gating strategy and quality control considerations. (a-b) Density dot plots of two representative samples. The gating hierarchy (shown with red arrows) for all samples was:

cells (R1) > singlets (R2) > live cells (R3) > cell specific markers (R4). Black arrow points out a few CD11b-/CD45+/7AAD- events in the sample depicted in **(b)** - this population is absent in the sample in **(a)**. We applied the wide gate (shown in **(b)**) whenever this additional population was observed. Please note that the final gate R4 in **(a)** includes all microglia cells (CD11b+/CD45+), while R4 in **(b)** also includes all microglia (CD11b+/CD45+) as well as the additional CD11b-/CD45+ peripheral immune cells population. Supplementary data 1 details the gating strategy for each sample. **(c-e)** The inclusion of peripheral immune cells through modified gating in some samples did not affect the detected microglia subsets. **(c)** t-SNE plot generated by using all of the cells from all samples - 9 microglia clusters (MG1-9) and 5 additional non-microglia clusters are present. **(d)** t-SNE plot generated from the samples with a gating strategy shown in **(a)**, that included all microglia but excluded the peripheral immune cells. Please note the presence of all 9 microglia clusters and the absence of the peripheral immune cell clusters (TC, BC, Mono). **(e)** t-SNE plot generated from the samples with a gating strategy shown in **(b)**, that included all microglia as well as the peripheral immune cell population present in some samples. Please note the presence of all 9 microglia clusters as well as the presence of the peripheral immune cell clusters (TC, BC, Mono). **(f-g)** The use of two different sorters did not affect the detected microglia subsets. The t-SNE plot generated from the cells sorted with Aria is shown in **(f)** and from the cells sorted on an Influx machine is shown in **(g)**. Please note the presence of all 9 microglia clusters (MG1-9) in both t-SNE plots. Abbreviations: FSC forwards scatter; SSC side scatter; 50K 50,000; 7AAD 7 Aminoactinomycin D that labels dead cells; AF488 AlexaFluor488 fluorochrome; AF647 AlexaFluor647 fluorochrome; MG microglia; TC T cells; BC B cells; GFAP glial fibrillary acidic protein; RBC erythrocytes.

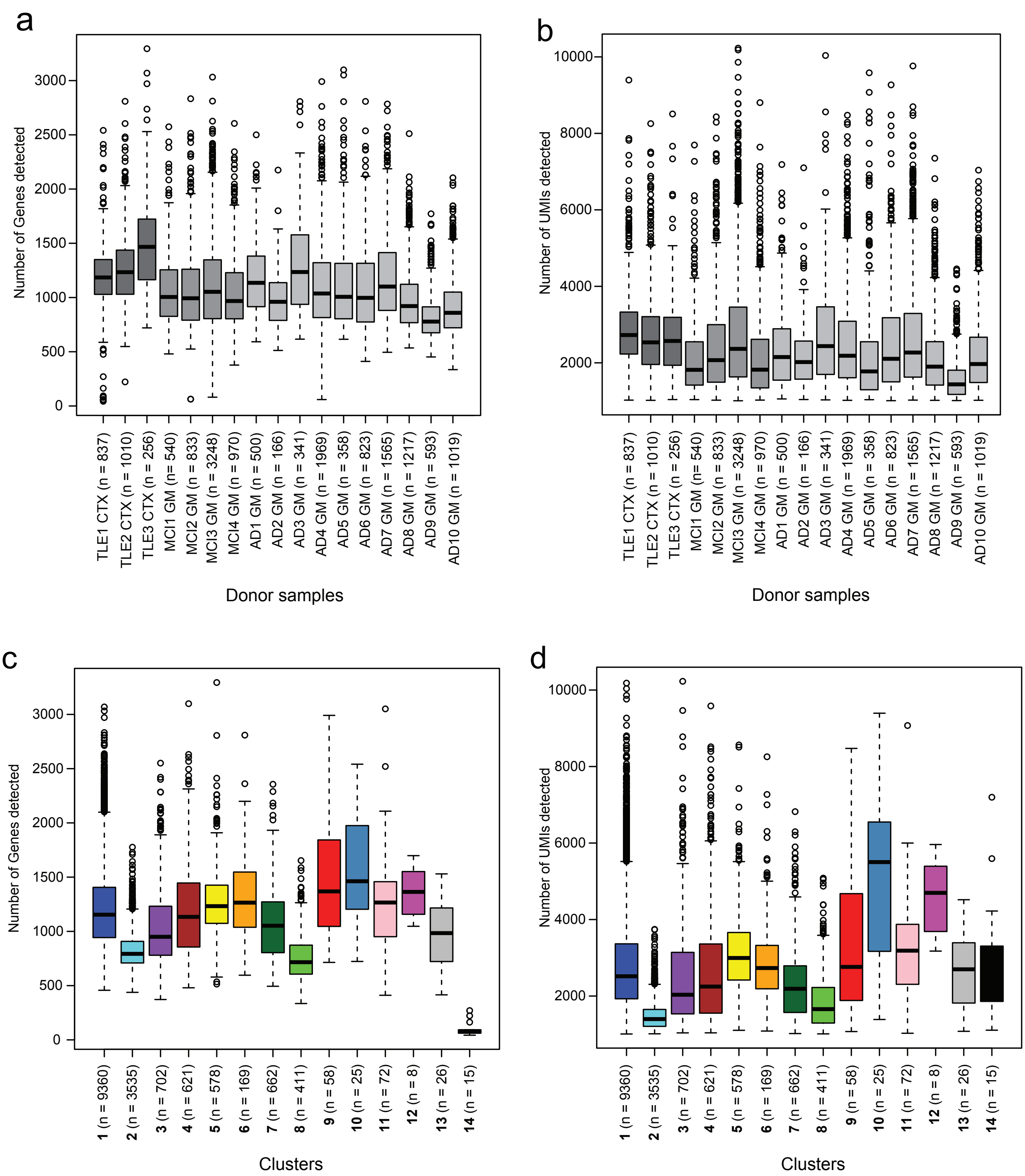

Supplementary figure 2. Data quality is comparable among the donors and microglial clusters. Quality control of the scRNA-seq dataset using the number of genes and unique molecular identifier (UMI) detected per cell as a quality measure. The box plots represent the median (middle line), 25%, and 75% percentiles. Whiskers extend to the most extreme cells no more than 1.5 times the interquartile range, and all cells more extreme than the whiskers are represented as circles. See **Supplementary data 1** for demographic and clinical data from each donor. The included donors and identified clusters are comparable in terms of number of detected genes (**(a)** and **(c)**) as well as the number of detected

UMIs (**(b)** and **(d)**). Abbreviations: TLE temporal lobe epilepsy; CTX cortex; AD Alzheimer's disease, MCI mild cognitive impairment; GM grey matter; UMI unique molecular identifier.

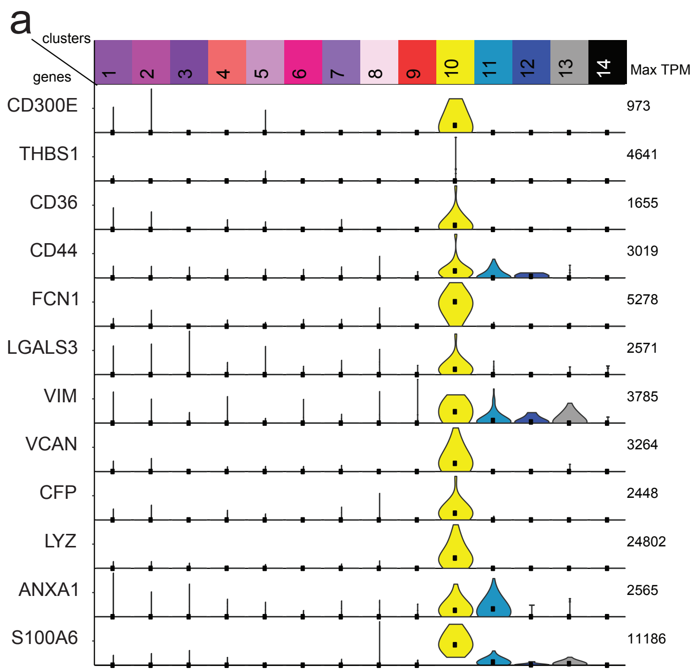

Supplementary figure 3. Identity of the myeloid clusters. (a) Violin plots representing the gene expression of genes that have been shown to be enriched in monocytes, when compared to microglia in a human study[1]. (**b)** Violin plots depicting the expression of genes that were found to be enriched in microglia when compared to monocytes in earlier mouse and human studies[1-4]. Abbreviations: TPM Transcripts Per Million.

Clusters	MCI1 GM	MCI2 GM	MCI3 GM	MCI4 GM	AD1 GM	AD2 GM	AD3 GM	AD4 GM	AD5 GM	AD6 GM	AD7 GM	AD8 GM	AD9 GM	AD10 GM	TLE1 CTX	TLE2 CTX	TLE3 CTX
1	59	513	1986	575	244	90	233	1280	194	526	928	785	323	683	290	472	179
2	100	180	687	342	137	35	80	406	134	218	407	286	119	234	27	140	3
3	19	22	228	3	10	11	2	106	3	4	128	75	7	69	3	12	
4	346	13	39	10	92	8	10	29	5	5	11	7	3	13		30	
5	2	6	22	8	3		10	14	13	2	11	6	8	6	343	114	10
6	5	5	11	4	1	1	4	5		7	12	2	2	2	56	22	30
7	2	65	109	4	10	10		24		2	10	39	122		71	170	27
8		14	153	23	2	7	2	75	4	48	48	7	4	11	5	6	2
9	7	13	4		1	3		24				4	1			1	
10								1			3	2	2		6	11	
11			1	1				1	4	9	4	4	1		21	21	5
12															3	5	
13			6			1		3	1	2	3		1	1	3	5	
14		2	2					1							9	1	

Donors

Supplementary figure 4. Distribution of cells in each cluster for each donor. Heatmap depicting the number of cells each donor contributed to a given cluster (rows) in each donor (columns). Cluster 1 and 2 are present in each sample and probably represent undifferentiated microglia clusters. Colors reflect the proportion of the total cells in a cluster that come from an individual donor. White = 0, pure yellow = 50%, and pure red = 100%. Abbreviations: MCI mild cognitive impairment; AD Alzheimer's disease; GM grey matter; TLE temporal lobe epilepsy; CTX cortex.

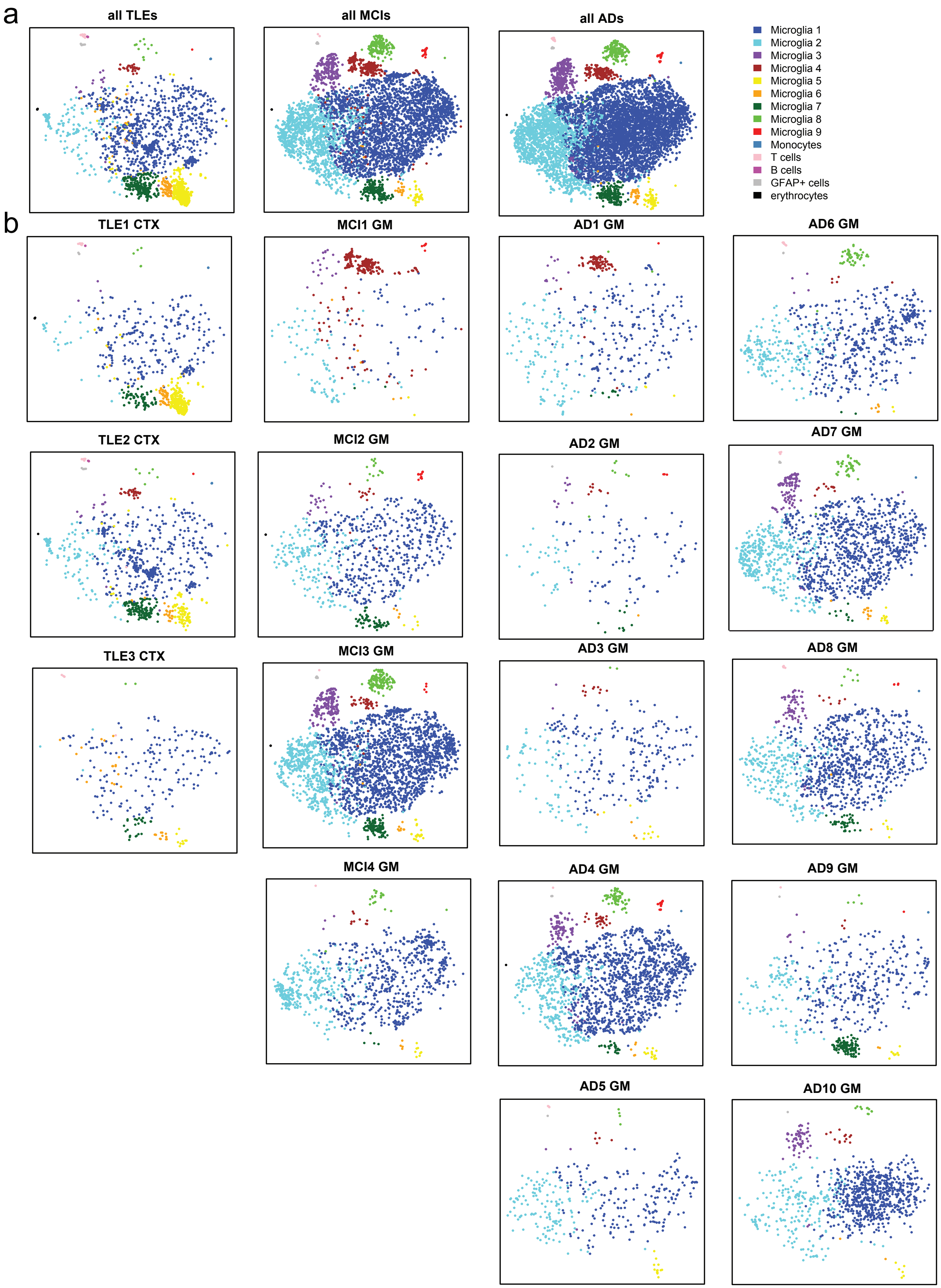

a
all TLEs
all MCIs
all ADs
Microglia 1
Microglia 2
Microglia 3
Microglia 4
Microglia 5
Microglia 6
Microglia 7
Microglia 8
Microglia 9
Monocytes
T cells
B cells
GFAP+ cells
erythrocytes
b
TLE1 CTX
MCI1 GM
AD1 GM
AD6 GM
TLE2 CTX
MCI2 GM
AD2 GM
AD7 GM
TLE3 CTX
MCI3 GM
AD3 GM
AD8 GM
MCI4 GM
AD4 GM
AD9 GM
AD5 GM
AD10 GM

Supplementary figure 5. The distribution of clusters is similar across pathologies and donors. t-SNE plots depicting the microglia population structure and distribution of the different clusters in the different pathology types (TLE, MCI, AD) in **(a)** and among the individual donors in **(b)**. Abbreviations: MCI mild cognitive impairment; AD Alzheimer's disease; GM grey matter; TLE temporal lobe epilepsy; CTX cortex.

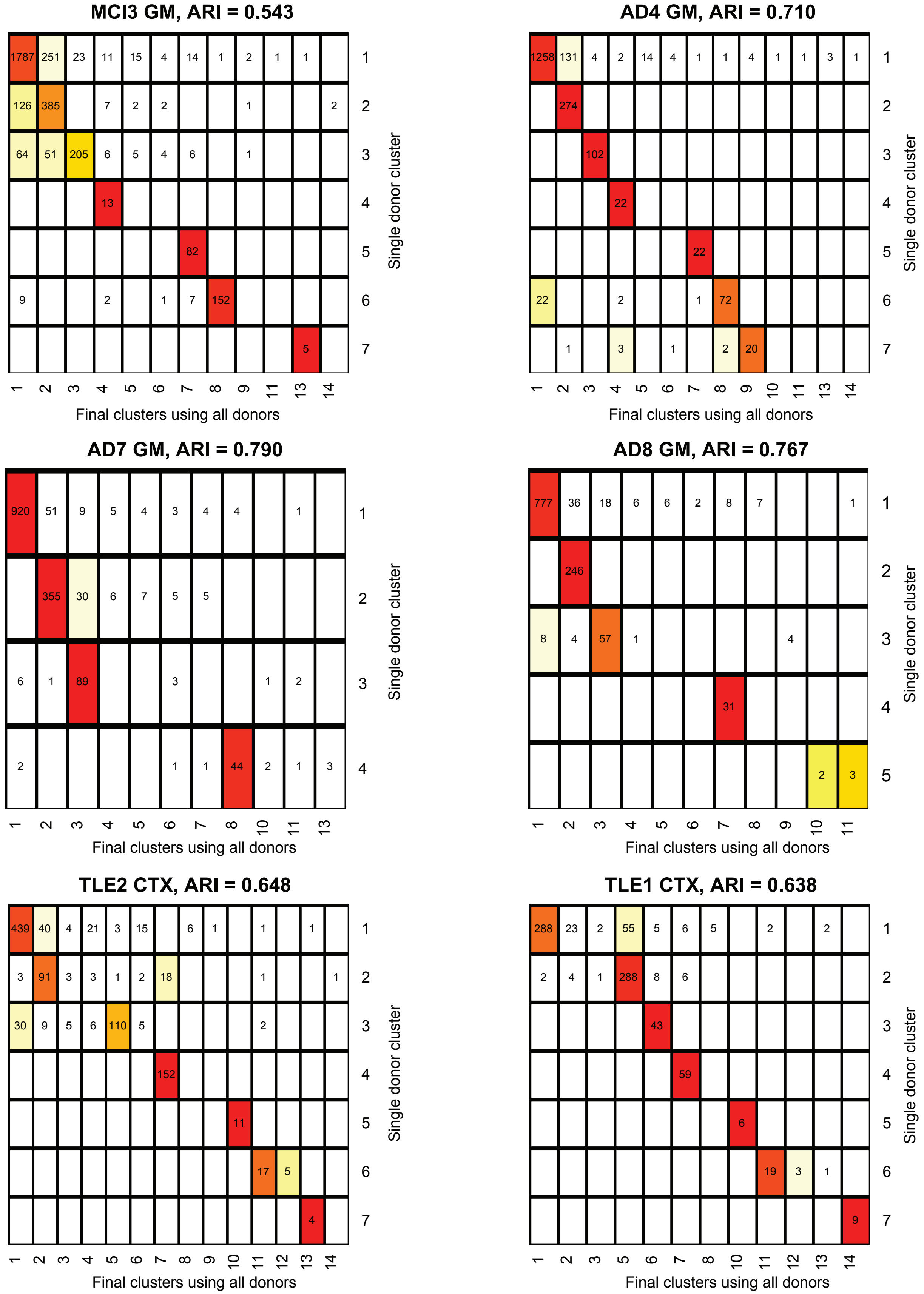

Supplementary figure 6. Assessment of cluster robustness using single donor datasets. Heatmaps depicting the number of cells in the different clusters generated on the whole dataset (columns) and on single donor datasets (rows) (see Supplementary data 1 for donor specifics). Please

note that for most donors, most cells are arranged along the diagonal suggesting good overlap between the overall clusters (generated using the full dataset) and the clusters generated based on the cells originating from a single donor. Colors reflect the proportion of the total cells in a cluster that come from an individual donor. White = 0, pure yellow = 50%, and pure red = 100%. The subject ID is listed at the top of each panel. Abbreviations: MCI mild cognitive impairment; AD Alzheimer’s disease; GM gray matter; TLE temporal lobe epilepsy; CTX cortex; ARI adjusted Rand index.

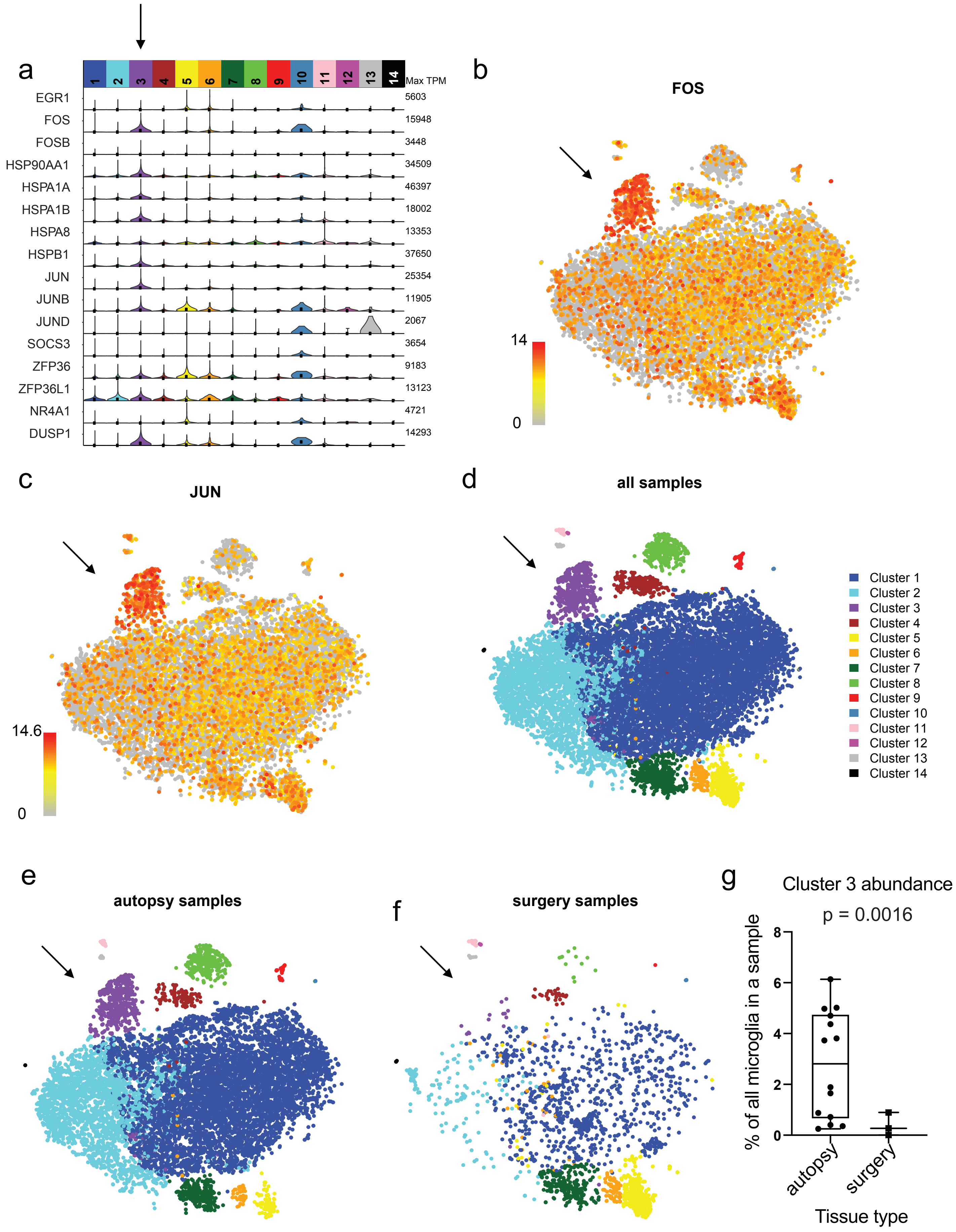

a
1 2 3 4 5 6 7 8 9 10 11 12 13 14
Max TPM
EGR1 5603
FOS 15948
FOSB 3448
HSP90AA1 34509
HSPA1A 46397
HSPA1B 18002
HSPA8 13353
HSPB1 37650
JUN 25354
JUNB 11905
JUND 2067
SOCS3 3654
ZFP36 9183
ZFP36L1 13123
NR4A1 4721
DUSP1 14293
b
FOS
14
0
c
JUN
14.6
0
d
all samples
Cluster 1
Cluster 2
Cluster 3
Cluster 4
Cluster 5
Cluster 6
Cluster 7
Cluster 8
Cluster 9
Cluster 10
Cluster 11
Cluster 12
Cluster 13
Cluster 14
e
autopsy samples
f
surgery samples
g
Cluster 3 abundance
p = 0.0016
% of all microglia in a sample
0
2
4
6
8
autopsy
surgery
Tissue type

Supplementary figure 7. Annotation of the signature prevalent in cluster 3. (a) Violin plots depicting the expression of the genes recently described to be induced by tissue dissociation and/or FACS in human skeletal muscle stem cells by van den Brink and colleagues[5]. Please note the strong upregulation of these genes in cluster 3 microglia, suggesting that they comprise cells in the state of distress. **(b-c)** Normalized gene expression levels projected on to the t-SNE plot for two genes known to be induced by cellular stress due to postmortem delay (FOS and JUN). Color gradient bars in **(b)** and **(c)** represent log2(TPM+1) which has been normalized, so that grey equals to 10th percentile expression value and red equals to maximum expressed value. Please note the enrichment of both of these genes in the cluster that we identified as cluster 3 **(d)**. **(e-f)** t-SNE plots generated using the cells from either the autopsy samples **(e)** or the surgery sample **(f)** only. Please not the lack of cluster 3 from surgery samples. **(g)** Quantification of the relative abundance of cluster 3 in autopsy (14 independent donor samples (MCI1 GM – MC4 GM, AD1 GM – AD10 GM)) versus surgery samples (3 independent donor samples (TLE1 CTX – TLE3 CTX)). Please note the reduction in cluster 3 abundance in the surgery samples. Unpaired t test with Welch's correction and two tailed P value was performed. The whiskers represent the minimum and the maximum of the data, the bounds of the box represent the 25th and 75th percentile of the data and the center line represents the mean. Source data are provided as a Source Data file. Abbreviations: FACS fluorescence activated cell sorting; MCI mild cognitive impairment; AD Alzheimer's disease; GM grey matter; TLE temporal lobe epilepsy; CTX cortex.

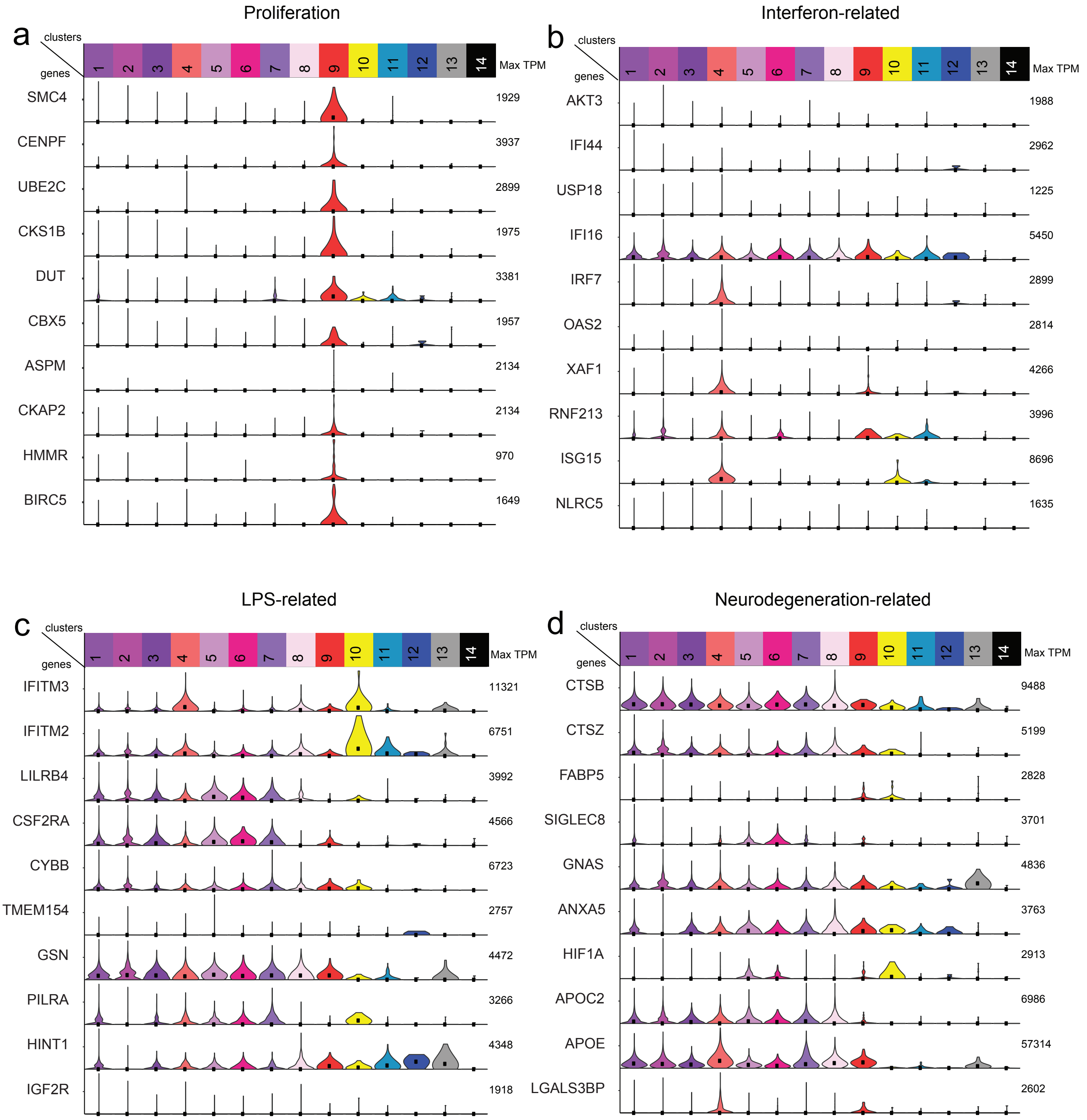

Supplementary figure 8. Cluster annotation based on previously published mouse studies[6]. Individual genes are present in each row, and each column represents a different cluster, as listed in the top banner of each panel. **(a)** Violin plots visualizing the genes associated with the proliferation related gene co-expression module. **(b)** Genes associated with the Interferon related gene co-expression module from the same study. **(c)** Violin plot showing the signature genes of the gene co-expression module associated with LPS response. **(d)** Neurodegeneration related genes. Please note that apart from the proliferation and interferon related gene sets the signatures identified in mouse fail to highlight a single human microglia subset. Abbreviations: TPM Transcripts Per Million; LPS lipopolysaccharide.

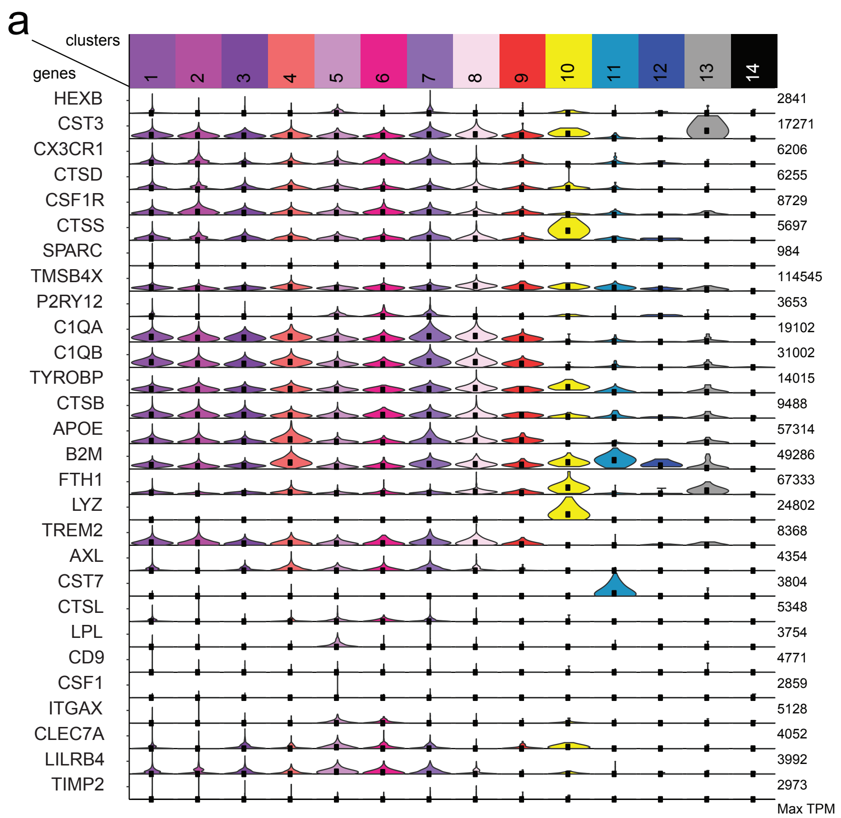
a
clusters
genes
1
2
3
4
5
6
7
8
9
10
11
12
13
14
HEXB
CST3
CX3CR1
CTSD
CSF1R
CTSS
SPARC
TMSB4X
P2RY12
C1QA
C1QB
TYROBP
CTSB
APOE
B2M
FTH1
LYZ
TREM2
AXL
CST7
CTSL
LPL
CD9
CSF1
ITGAX
CLEC7A
LILRB4
TIMP2
2841
17271
6206
6255
8729
5697
984
114545
3653
19102
31002
14015
9488
57314
49286
67333
24802
8368
4354
3804
5348
3754
4771
2859
5128
4052
3992
2973
Max TPM

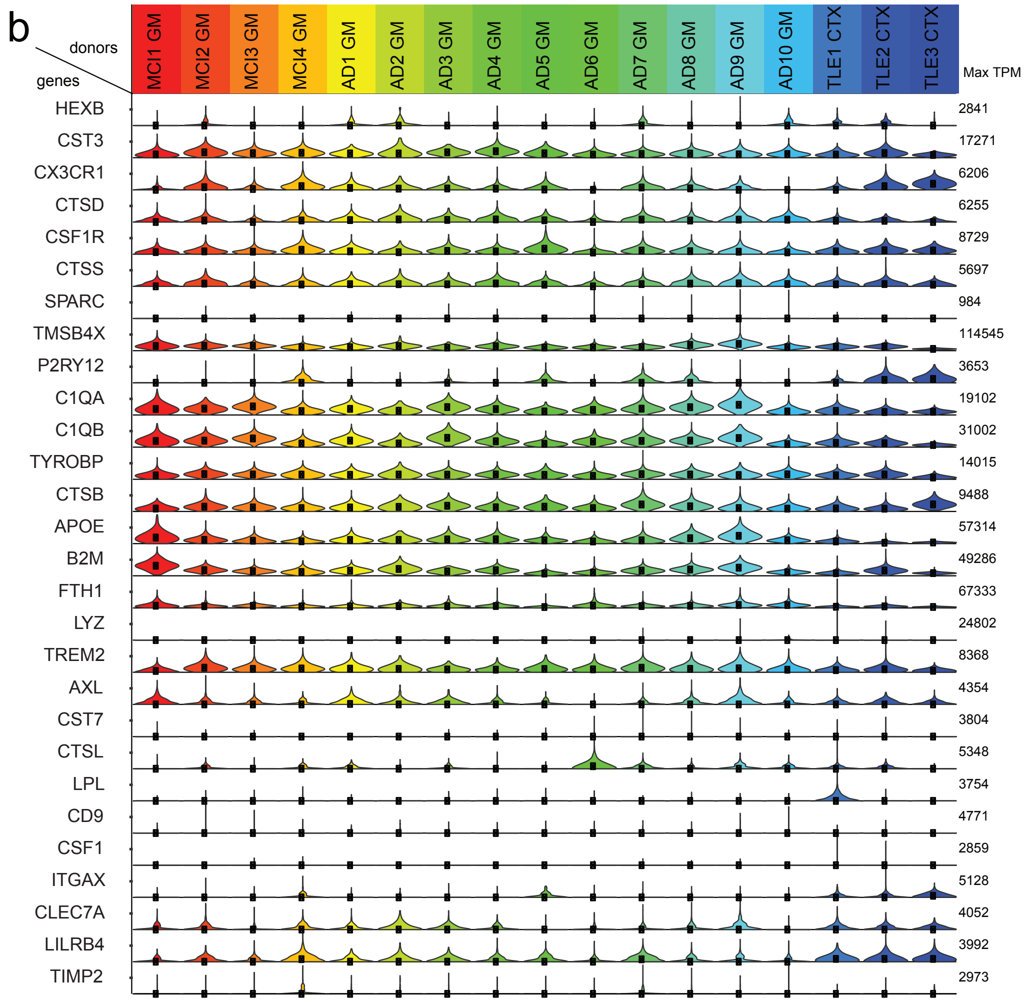
b
donors
genes
MCI1 GM
MCI2 GM
MCI3 GM
MCI4 GM
AD1 GM
AD2 GM
AD3 GM
AD4 GM
AD5 GM
AD6 GM
AD7 GM
AD8 GM
AD9 GM
AD10 GM
TLE1 CTX
TLE2 CTX
TLE3 CTX
Max TPM
HEXB
CST3
CX3CR1
CTSD
CSF1R
CTSS
SPARC
TMSB4X
P2RY12
C1QA
C1QB
TYROBP
CTSB
APOE
B2M
FTH1
LYZ
TREM2
AXL
CST7
CTSL
LPL
CD9
CSF1
ITGAX
CLEC7A
LILRB4
TIMP2
2841
17271
6206
6255
8729
5697
984
114545
3653
19102
31002
14015
9488
57314
49286
67333
24802
8368
4354
3804
5348
3754
4771
2859
5128
4052
3992
2973

Supplementary figure 9. Cluster and donor wise expression of the DAM signature genes. (a) Violin plots depicting the expression pattern of the homeostatic and mouse Disease Associated Microglia (DAM)[7] signature genes in the different human microglia subsets. Each row presents the expression of one gene, as listed on the left. Each column is one of our 14 clusters. **(b)** Violin plots showing the expression pattern of the homeostatic and DAM signature genes (rows) in the microglia of different donors (columns) that participated in our study. Please note the general expression of the DAM signature genes across the different human microglia subsets **(a)** and across the different donors **(b)**. Abbreviations: TPM Transcripts Per Million; MCI mild cognitive impairment; AD Alzheimer's disease; GM grey matter; TLE temporal lobe epilepsy; CTX cortex.

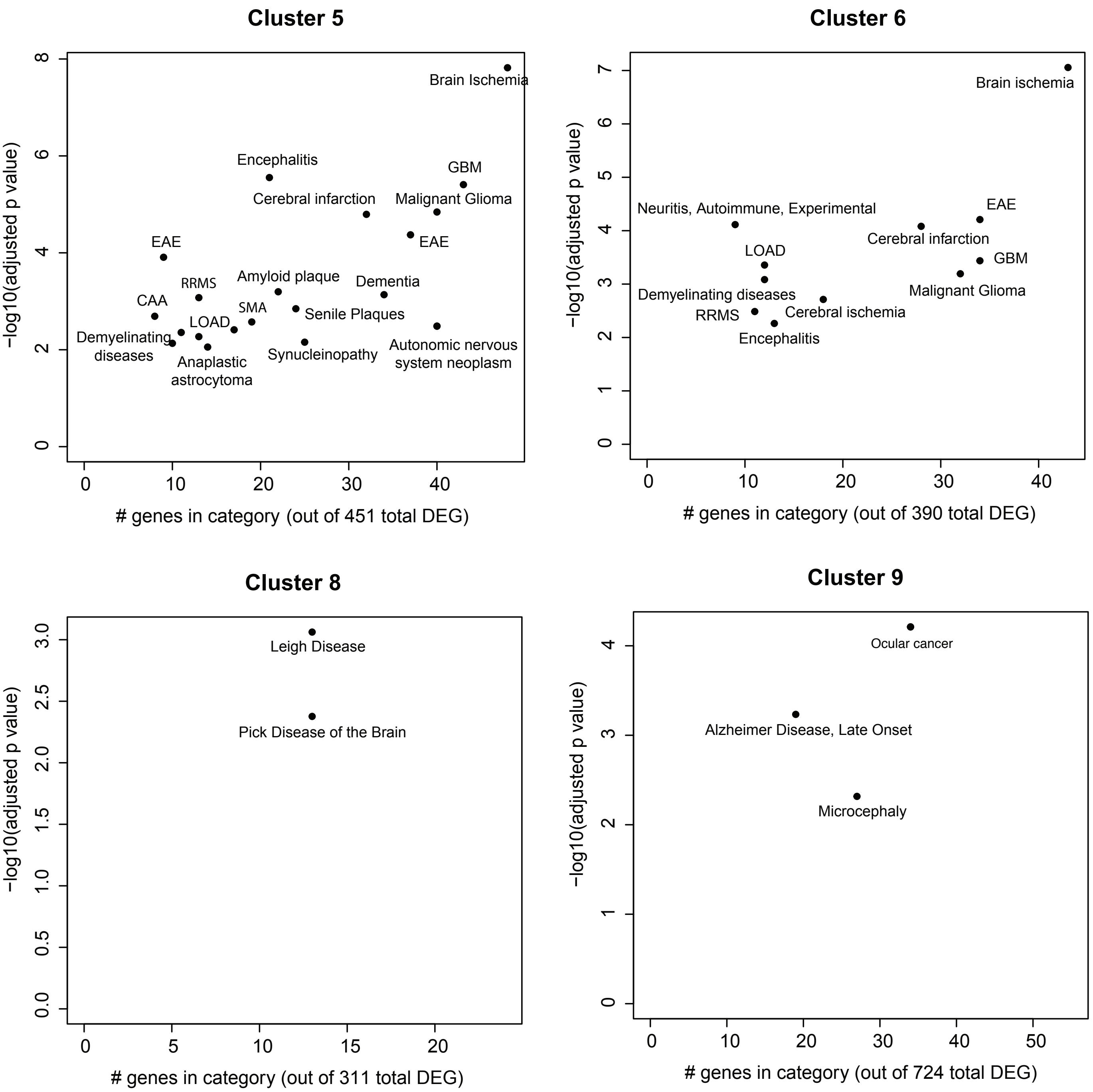

Supplementary figure 10. Disease enrichment in microglial subsets. Scatter plots depicting brain related diseases that are significantly enriched with genes that are marker genes for different clusters. The disease associated gene sets come from the disease ontology database (http://disease-ontology.org/) and the signature gene sets of each microglia cluster, listed in Supplementary Data 10. The significance of the enrichment (-log(pvalue)) is represented in the y axis, and the number of overlapping genes is reported on the x axis. Abbreviation: EAE experimental allergic encephalomyelitis; LOAD late onset Alzheimer’s disease; GBM glioblastoma multiforme; RRMS relapsing remitting multiple sclerosis; EAE experimental autoimmune encephalomyelitis; CAA cerebral amyloid angiopathy; SMA spinal muscular atrophy; DEG differentially expressed genes.

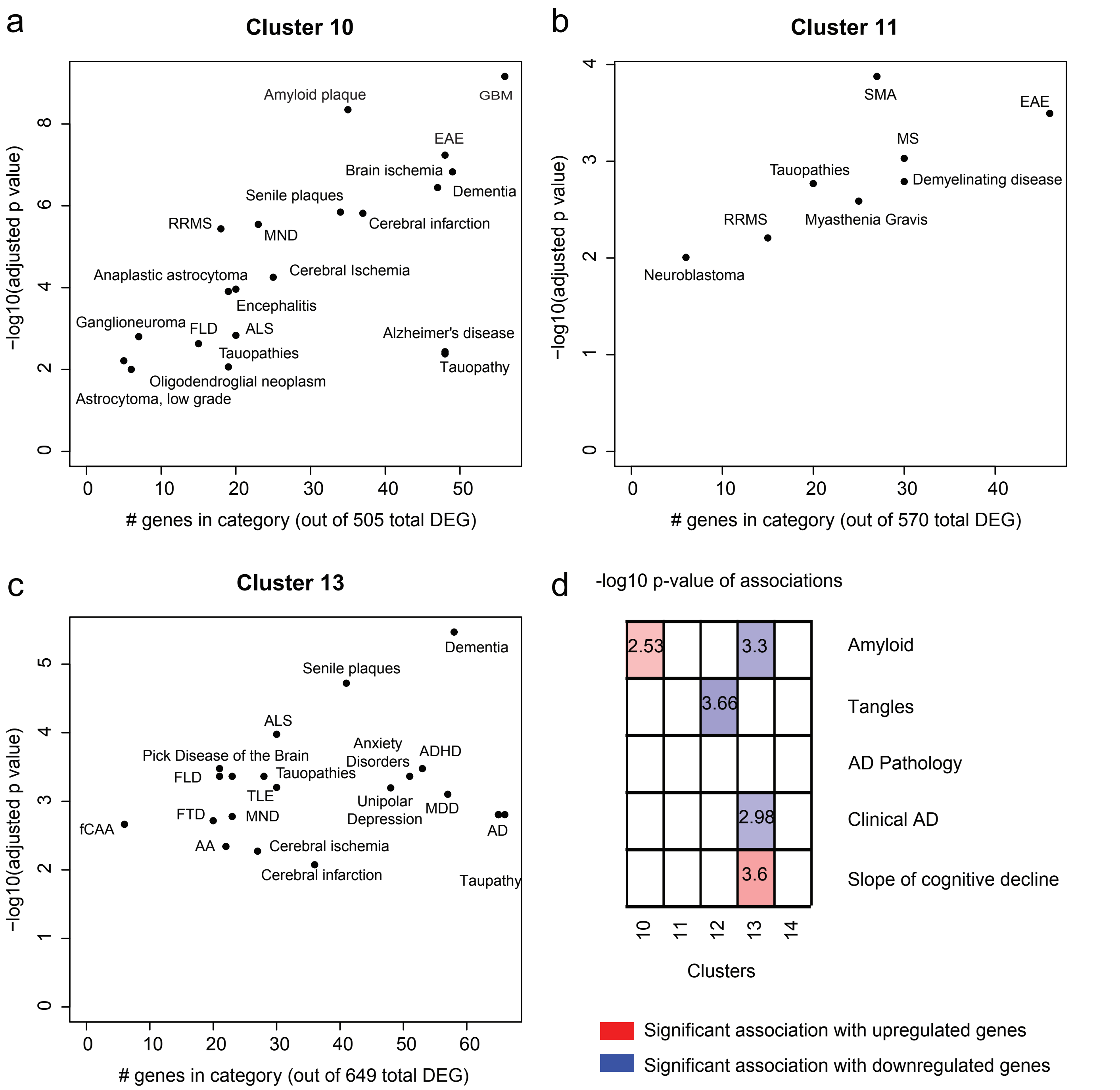

Supplementary figure 11. Disease enrichment among the non-microglial cell subsets. (a-c) Scatter plots depicting brain related diseases that are significantly enriched with the different clusters based on an enrichment analysis between disease associated gene sets from the disease ontology database (http://disease-ontology.org/) and the signature gene sets of each microglia cluster. The significance of the enrichment (-log(pvalue)) is represented in the y axis, and the number of overlapping genes is reported on the x axis. **(d)** Panel reporting the result of enrichment analyses between the genes defining the non-microglial clusters and those genes that are associated with pathological or clinical traits found in the aging human brain in the ROS and MAP cohorts. p-values are shown for those cluster/trait combinations where they are significant, and the color of each box is related to the strength of the association. The adjusted p-values shown here are obtained from the rank-rank hypergeometric tests. Abbreviation: DEG differentially expressed genes; CIDP Chronic Inflammatory Demyelinating Polyneuropathy; EAE experimental allergic encephalomyelitis; FTD frontotemporal dementia; GBM glioblastoma multiforme; RRMS relapsing-remitting multiple sclerosis; ALS amyotrophic lateral sclerosis; HD Huntington disease; AD Alzheimer’s disease; LWD Lewy body

disease; TLE temporal lobe epilepsy; ADHD attention deficit hyperactivity disorder; CJD Creutzfeldt-Jakob Disease; fCAA familial cerebral amyloid angiopathy; SMA spinal muscular atrophy; RRMS relapsing remitting multiple sclerosis; MND motor neuron disease; EAE experimental autoimmune encephalomyelitis; MS multiple sclerosis; AA amyloid angiopathy; MDD major depressive disorder; FLD frontal lobe dementia; DEG differentially expressed genes.

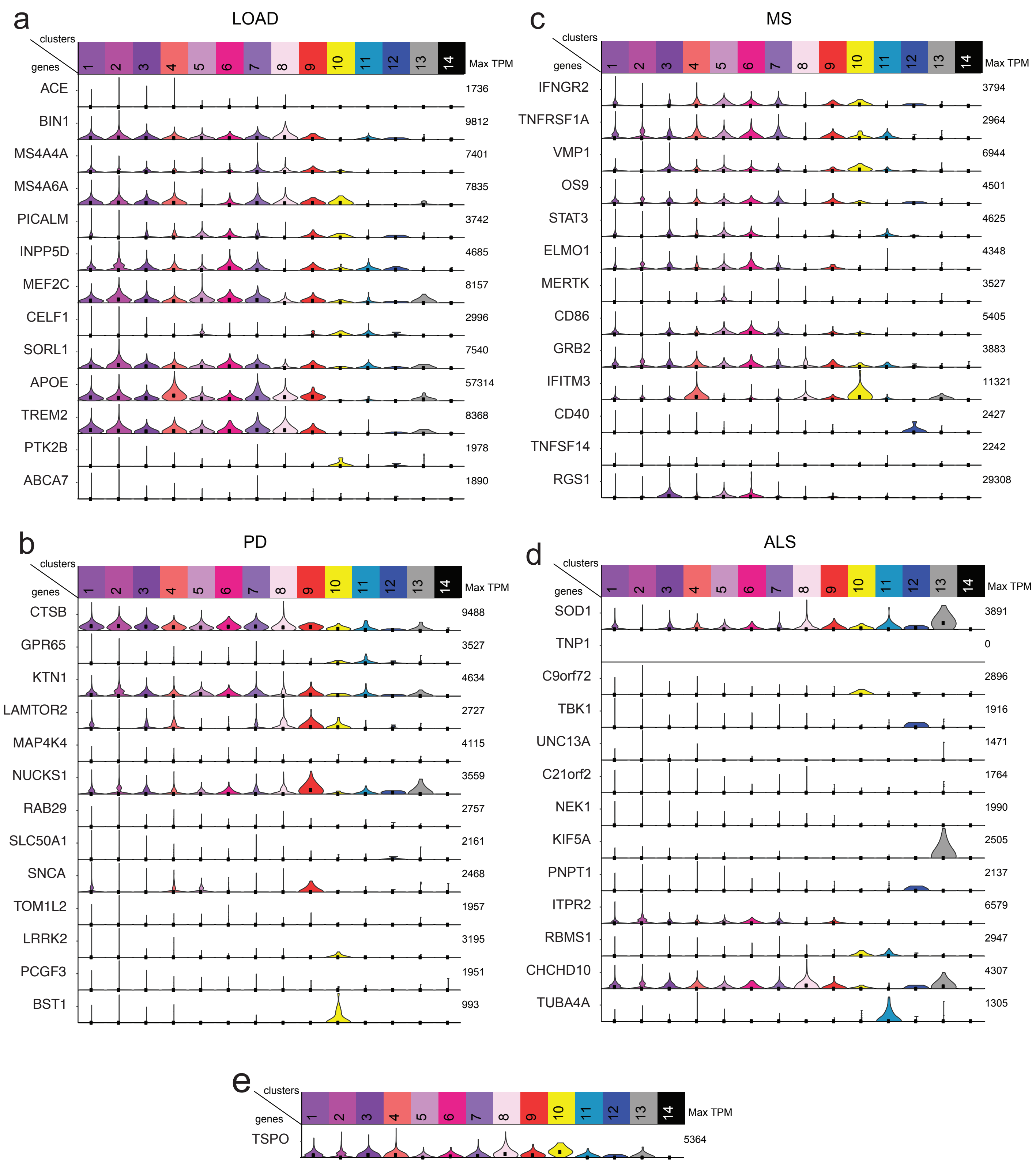

Supplementary figure 12. The expression of neurodegenerative disease susceptibility genes. Violin plots showing the distribution of expression levels of genes associated with LOAD **(a)**, PD **(b),** MS **(c)** and ALS **(d)** (from the GWAS catalog (https://www.ebi.ac.uk/gwas/)), respectively. Additionally, **(e)** shows the cluster-wise expression level of TSPO, a marker used to monitor microglia activation with positron emission tomography (PET). Only those genes are shown that had a detectable level of expression in at least one of the clusters. Abbreviations: TPM Transcripts Per Million; LOAD late onset Alzheimer's disease; MS multiple sclerosis; PD Parkinson's disease; ALS amyotrophic lateral sclerosis.

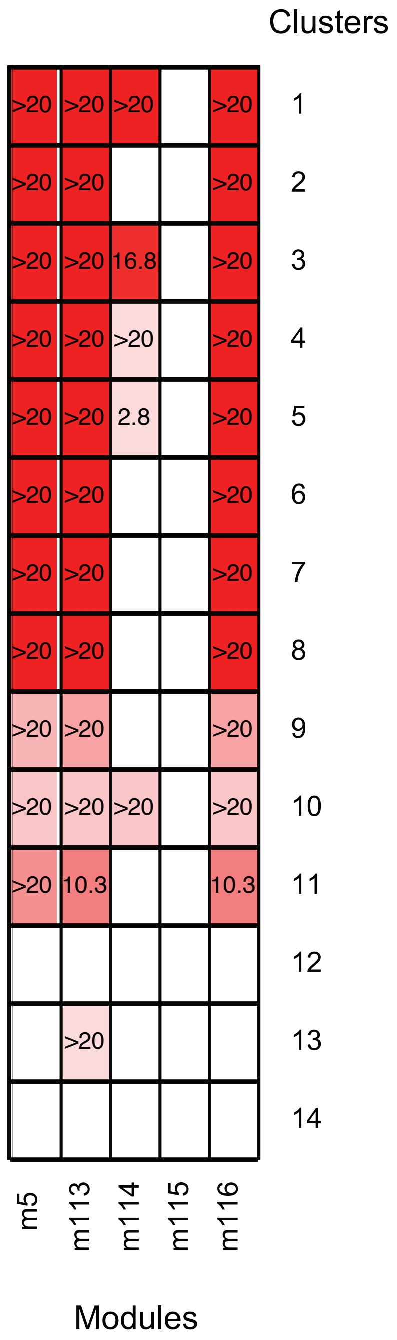

Supplementary figure 13. Association with gene co-expression modules of the human brain. Heatmap showing the enrichment for the genes defining a given cell cluster (rows) in each microglia module (column) defined from bulk DLPFC data. We have previously identified microglia related gene co-expression modules in the human dorsolateral prefrontal cortex[8]. Color coding: FDR-adjusted p-values (log-10 transformed) are scaled by row, with pure red = maximum value over all modules. White values indicate non-significance (FDR-adjusted p-values > 0.05). The numbers in the boxes represent the -log (adjusted p-value) from standard gene-set enrichment analysis. Abbreviation: DLPFC dorsolateral prefrontal cortex; m stands for module.

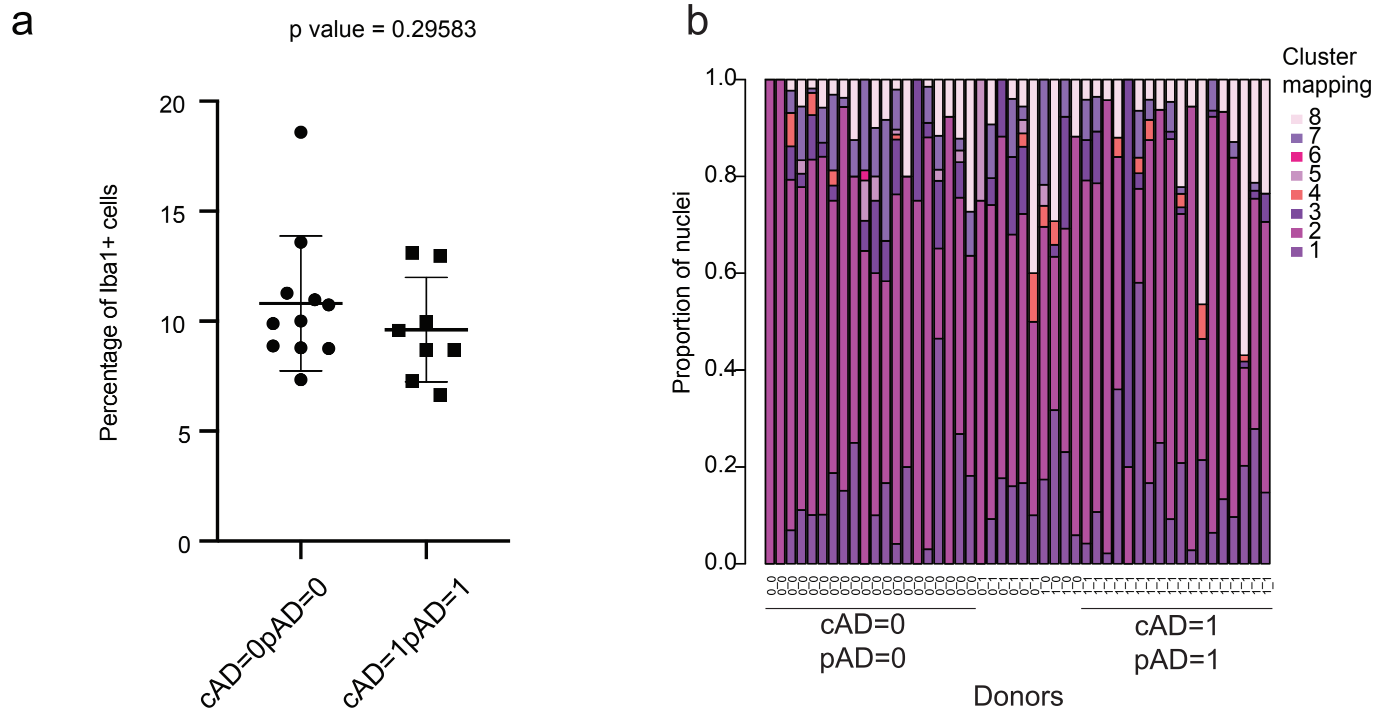

Supplementary figure 14. Replication of cluster 7 association with AD. In **(a)** we present a dot plot comparing the frequency of IBA1^{+} cells within all the cells in DLPFC tissue sections from New York Brain Bank subjects with both AD dementia and a pathological diagnosis of AD (cAD=1, pAD=1; n=8) to that found in subjects who fulfill neither of these diagnostic criteria (cAD=0, pAD=0; n=11). These data were collected using CellProfiler. Every dot is an individual donor (see Supplementary Data 9). Overlaid on the dot plot, data are also presented as mean values +/- SD. The statistical test used was an unpaired t test with a two tailed p value. See Supplementary Data 9 for demographics of the donors and Source Data file for raw data. Source data are provided in the Source Data file. In **(b)**, we present the repurposed single nucleus RNA sequencing data[9] and assigned each microglial nuclear transcriptomic profile to one of our nine microglial clusters using the cluster-defining gene sets. In this panel, each column presents the proportion of microglial nuclei in each cluster in one individual. The clusters are color coded following the key on the right (introduced in **Figure 2a**); none of the nuclei map to cluster 9. Subjects meeting one or the other set of criteria cAD=1, pAD=1 or cAD=0, pAD=0 are denoted below the x axis. Abbreviations: cAD clinical diagnosis of AD dementia; pAD pathological diagnosis of AD dementia.

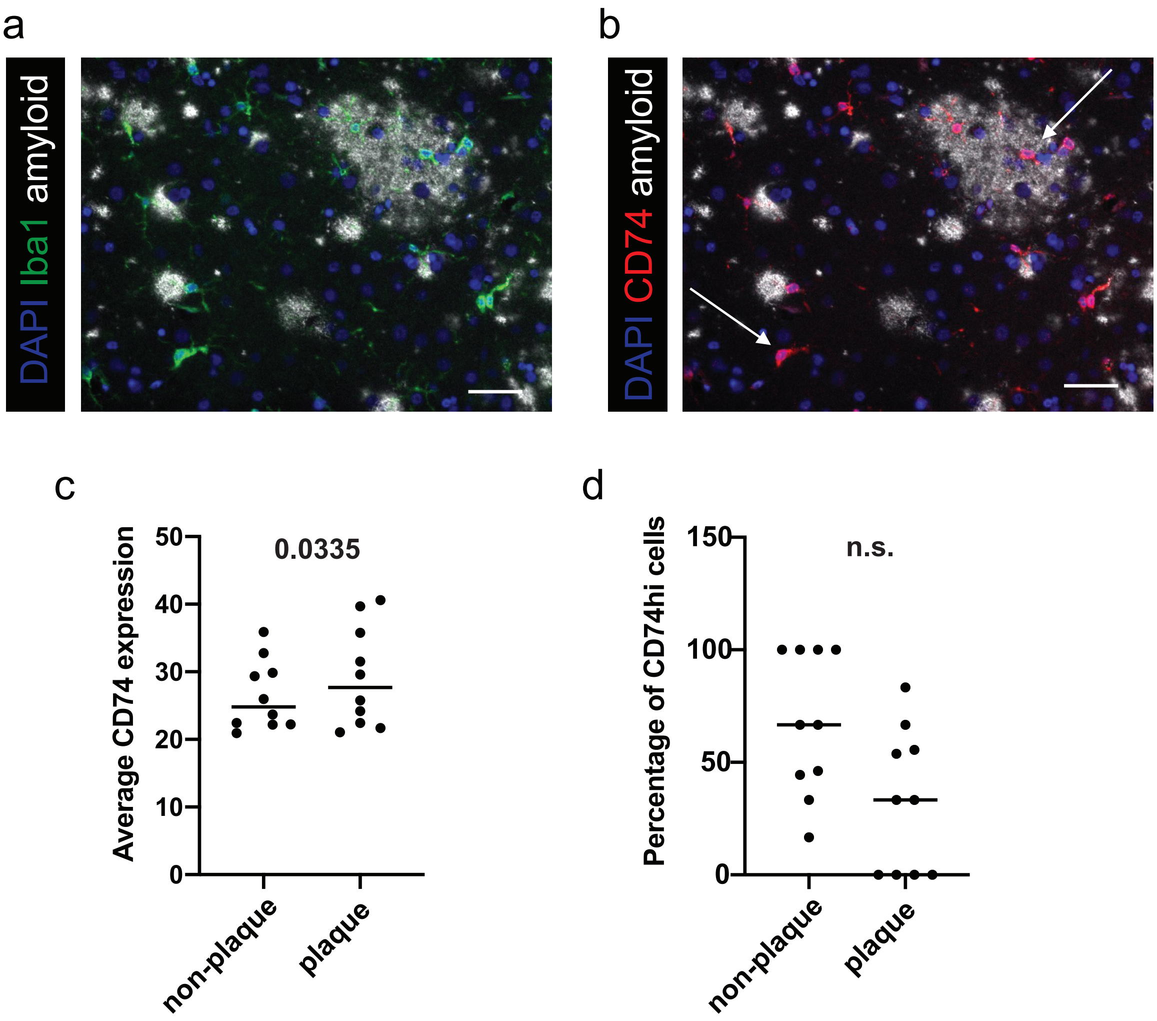

Supplementary figure 15. In situ analysis of cluster 7 distribution as it relates to topology. Cluster 7 microglia cells are not preferentially associated with amyloid plaques. The distribution of cluster 7 cells in relationship to amyloid plaques was assessed using immunohistochemistry, fluorescence microscopy and automated image analysis (CellProfiler). **(a)** and **(b)** are representative photomicrographs showing the stainings for amyloid, Iba1 and CD74. Bars in the lower right corners of the images represent 50 µm. These experiments were performed in ten individual donors. In each donor 15-20 images were captured in the grey matter of the DLPFC and analyzed using IHC and automated image analysis. Cluster 7 cells were identified based on their CD74 high expression. **(c)** Dot plots depicting the average CD74 expression in microglia that are either outside of a plaque (non-plaque), or associated with a plaque (plaque) in 10 donors. Each dot represents a donor. For significance testing a paired, two-tailed t test was performed. The center line represents the median. **(d)** Dot plots depicting the percentage of CD74hi (cluster 7) microglia either outside of a plaque (non-plaque), or associated with a plaque (plaque) in 10 donors. Each dot represents a donor. For significance testing a paired, two-tailed t test was performed. The center line represents the median. Source data for **(c)** and **(d)** are provided in the Source Data fileAbbreviation: DAPI 4′,6-diamidino-2-phenylindole, a fluorescent DNA stain; Iba1 Ionized calcium binding adaptor molecule 1, a general microglia marker; n.s. not significant.

Supplementary references

1. Galatro, T.F.*, et al.* Transcriptomic analysis of purified human cortical microglia reveals age-associated changes. *Nat Neurosci* **20**, 1162-1171 (2017).
2. Hickman, S.E.*, et al.* The microglial sensome revealed by direct RNA sequencing. *Nat Neurosci* **16**, 1896-1905 (2013).
3. Chiu, I.M.*, et al.* A neurodegeneration-specific gene-expression signature of acutely isolated microglia from an amyotrophic lateral sclerosis mouse model. *Cell Rep* **4**, 385-401 (2013).
4. Butovsky, O.*, et al.* Identification of a unique TGF-beta-dependent molecular and functional signature in microglia. *Nat Neurosci* **17**, 131-143 (2014).
5. van den Brink, S.C.*, et al.* Single-cell sequencing reveals dissociation-induced gene expression in tissue subpopulations. *Nat Methods* **14**, 935-936 (2017).
6. Friedman, B.A.*, et al.* Diverse Brain Myeloid Expression Profiles Reveal Distinct Microglial Activation States and Aspects of Alzheimer's Disease Not Evident in Mouse Models. *Cell Rep* **22**, 832-847 (2018).
7. Keren-Shaul, H.*, et al.* A Unique Microglia Type Associated with Restricting Development of Alzheimer's Disease. *Cell* **169**, 1276-1290 e1217 (2017).
8. Patrick, E., et al. A cortical immune network map identifies a subset of human microglia involved in Tau pathology. https://doi.org/10.1101/234351 (2017).
9. Mathys, H.*, et al.* Single-cell transcriptomic analysis of Alzheimer's disease. *Nature* **570**, 332–337 (2019).
