# Supplementary material for: Single cell RNA sequencing of human microglia uncovers a subset associated with Alzheimer’s disease
Source: Nat Commun. 2020 Nov 30;11:6129. doi: 10.1038/s41467-020-19737-2 (PMC7704703; doi:10.1038/s41467-020-19737-2)
Supplement: Supplementary file 2 — Description of Additional Supplementary Files [file 41467_2020_19737_MOESM2_ESM.pdf]

Description of Additional Supplementary Files

File Name: Supplementary Data 1
Description: Demographic, clinical and neuropathological overview of the included samples.

File Name: Supplementary Data 2
Description: Sequencing metrics of the profiled samples. CellRanger quality control output file following 10x Chromium single cell RNA sequencing.

File Name: Supplementary Data 3
Description: Cluster wise mean gene expression (TPM) for each detected gene. Columns represent the identified clusters (cluster 1 to 14) and the detected genes are shown in rows.

File Name: Supplementary Data 4
Description: Cluster wise fraction of expression for each detected gene. Columns represent the identified clusters (cluster 1 to 14) and the detected genes are shown in rows. For each cluster, the values represent the fraction of cells in which a given gene was detected (>0 TPM).

File Name: Supplementary Data 5
Description: Cluster wise signature gene sets. Genes found to be upregulated in each cluster, ordered by the number of pairwise comparisons between clusters in which they came up as significantly upregulated. Importantly, for a gene to be included for a cluster, it must not be downregulated in that cluster with respect to any other clusters.

File Name: Supplementary Data 6
Description: Transcriptional regulation of the different microglia subsets. For each cluster a signature gene set was identified (top 50 gene for each cluster based on the list in Supplementary data 5). The enrichment of the promoters of the genes belonging to these gene sets in transcription factor matrices was tested using PASTAA.

File Name: Supplementary Data 7
Description: Functional annotation of the different microglia subsets. For each cluster a signature gene set was identified (top 50 gene for each cluster based on the list in Supplementary data 5). These sets were submitted for pathway analysis in REACTOME.

File Name: Supplementary Data 8
Description: Functional annotation of the different microglia subsets using other mouse and human microglia single cell RNA sequencing datasets. Tab 1 contains the comparison to the mouse dataset shown in Keren-Shaul et al. (2017), tab 2 contains the comparison to the mouse dataset in Mathys et al. (2017) and tab 3 has the comparison to the human dataset described in Sankowski et al. (2019).

File Name: Supplementary Data 9
Description: Demographic and clinicopathological overview of the samples used for the in situ confirmation studies.

File Name: Supplementary Data 10
Description: Disease ontology analysis of the identified clusters.

File Name: Supplementary Data 11
Description: Analysis of the association of the cluster signatures with clinicopathological traits in the ROSMAP cohort.

File Name: Supplementary Data 12
Description: Analysis of the association of the microglia cluster signatures with microglia enriched cortical gene co-expression modules identified in the ROSMAP cohort (Mostafavi et al. (2018)).

File Name: Supplementary Data 13
Description: Mapping the nuclei from the Mathys et al. (2019) study to the identified microglia clusters. Each column represents a donor and their pathological and clinical classification is shown in column headers. In rows the cluster wise proportions of mapped nuclei are shown. Please note that no nucleus mapped to cluster 9. Yes_dementia cAD=1; No_dementia cAD=0; Yes_pathology pAD=1; No_pathology pAD=0.

File Name: Supplementary Data 14
Description: Cell by gene counts matrix.

File Name: Supplementary Data 15
Description: Cell annotations.

File Name: Supplementary Data 16
Description: Cell by gene mean z score matrix.
