# Supplementary material for: Single cell RNA sequencing of human microglia uncovers a subset associated with Alzheimer’s disease
Source: Nat Commun. 2020 Nov 30;11:6129. doi: 10.1038/s41467-020-19737-2 (PMC7704703; doi:10.1038/s41467-020-19737-2)
Supplement: Supplementary file 19 — Reporting Summary [file 41467_2020_19737_MOESM19_ESM.pdf]

nature research

Corresponding author(s): De Jager, Philip L

Last updated by author(s): 10/04/2020

Reporting Summary

Nature Research wishes to improve the reproducibility of the work that we publish. This form provides structure for consistency and transparency in reporting. For further information on Nature Research policies, see Authors & Referees and the Editorial Policy Checklist .

Statistics

For all statistical analyses, confirm that the following items are present in the figure legend, table legend, main text, or Methods section.

n/a	Confirmed	
☐	☒	The exact sample size (*n*) for each experimental group/condition, given as a discrete number and unit of measurement
☐	☒	A statement on whether measurements were taken from distinct samples or whether the same sample was measured repeatedly
☐	☒	The statistical test(s) used AND whether they are one- or two-sided<br>*Only common tests should be described solely by name; describe more complex techniques in the Methods section.*
☒	☐	A description of all covariates tested
☐	☒	A description of any assumptions or corrections, such as tests of normality and adjustment for multiple comparisons
☐	☒	A full description of the statistical parameters including central tendency (e.g. means) or other basic estimates (e.g. regression coefficient) AND variation (e.g. standard deviation) or associated estimates of uncertainty (e.g. confidence intervals)
☐	☒	For null hypothesis testing, the test statistic (e.g. *F*, *t*, *r*) with confidence intervals, effect sizes, degrees of freedom and *P* value noted<br>*Give P values as exact values whenever suitable.*
☒	☐	For Bayesian analysis, information on the choice of priors and Markov chain Monte Carlo settings
☒	☐	For hierarchical and complex designs, identification of the appropriate level for tests and full reporting of outcomes
☐	☒	Estimates of effect sizes (e.g. Cohen's *d*, Pearson's *r*), indicating how they were calculated

Our web collection on statistics for biologists contains articles on many of the points above.

Software and code

Policy information about availability of computer code

Data collection	CellRanger software (from 10x Genomics) was used to align and quantify single-cell RNA-seq transcripts
Data analysis	R statistical software was used to analyze data, and all custom code will be made available accompanying the publication

For manuscripts utilizing custom algorithms or software that are central to the research but not yet described in published literature, software must be made available to editors/reviewers. We strongly encourage code deposition in a community repository (e.g. GitHub). See the Nature Research guidelines for submitting code & software for further information.

Data

Policy information about availability of data

All manuscripts must include a data availability statement. This statement should provide the following information, where applicable:

- Accession codes, unique identifiers, or web links for publicly available datasets
- A list of figures that have associated raw data
- A description of any restrictions on data availability

Single-cell RNA-seq data is available through Synapse (synapse.org sublink is generated). All figures in the paper are based on this RNA-seq data.

Field-specific reporting

Please select the one below that is the best fit for your research. If you are not sure, read the appropriate sections before making your selection.

☒ Life sciences ☐ Behavioural & social sciences ☐ Ecological, evolutionary & environmental sciences

For a reference copy of the document with all sections, see nature.com/documents/nr-reporting-summary-flat.pdf

Life sciences study design

All studies must disclose on these points even when the disclosure is negative.

Sample size	Sample sizes were not calculated ahead of time, but we required at least 10 donors in total to assure that a sufficient degree of cell type heterogeneity would be investigated. As with most single-cell RNA-seq studies, the number of cells per donor is determined by the protocol, and is only roughly controllable.
Data exclusions	Cells with fewer than 1000 transcripts (Unique Molecular Identifiers) were excluded from subsequent analyses. This threshold, a standard for low-depth sequencing, was chosen to ensure that downstream clustering and gene expression identification were not skewed by cells with poor detection rates.
Replication	In the manuscript we replicated the microglia clusters in an independent human single cell RNAs equencing dataset generated by our collaborators. We also replicated our findings in a published single nucleus RNA sequencing dataset.
Randomization	The identification of clusters does not require the specification of sample groups, so no randomization was performed. Potential batch-related effects were accounted for in the clustering by regressing the count data against batch ID and clustering on the residuals, as described in the text.
Blinding	Blinding was not relevant to the study, since the algorithm for identifying clusters does not take any sample or donor metadata into account.

Reporting for specific materials, systems and methods

We require information from authors about some types of materials, experimental systems and methods used in many studies. Here, indicate whether each material, system or method listed is relevant to your study. If you are not sure if a list item applies to your research, read the appropriate section before selecting a response.

Materials & experimental systems

n/a	Involved in the study	
☐	☒	Antibodies
☒	☐	Eukaryotic cell lines
☒	☐	Palaeontology
☒	☐	Animals and other organisms
☐	☒	Human research participants
☒	☐	Clinical data

Methods

n/a	Involved in the study	
☒	☐	ChIP-seq
☐	☒	Flow cytometry
☒	☐	MRI-based neuroimaging

Antibodies

Antibodies used	Iba1 (Wako, CatNo: 019-19741, LotNo: WDJ3047); CD45 (Novus, CatNo: NB500-319, LotNo: 527521); ISG15 (Proteintech, CatNo: 15981-1-AP, LotNo: n/a); CD83 (BioLegend, CatNo: 305302, LotNo: B201387); CD74 (BioLegend, CatNo: 326802, LotNo: B264972); PCNA (Invitrogen, CatNo: 13-3900, LotNo: TG268310); AlexaFluor488 anti-human CD11b (BioLegend, CatNo: 101217, LotNo: many different lots); AlexaFluor647 anti-human CD45 (BioLegend, CatNo: 304056, LotNo: many different lots).
Validation	The anti Iba1 from Wako is an extensively used antibody to detect microglia in the brain. BioLegend's anti-human CD11b and CD45 antibodies are used by us since several years, and the transcriptomic analysis of cells sorted using these two antibodies confirmed that they label myeloid cells which in the brain is primarily microglia (PMID: 29416036). The specificity of the unconjugated anti-human CD45 antibody from Novus (CatNo: NB500-319, clone: MEM-28) has been validated on human peripheral blood (https://www.novusbio.com/products/cd45-antibody-mem-28_nb500-319). The Proteintech antibody (anti ISG15) was extensively validated by the vendor which efforts are documented on their website (https://www.ptglab.com/products/ISG15-Antibody-15981-1-AP.htm#validation). The anti CD83 antibody was validated on human monocyte derived dendritic cells by the vendor (https://sandbox.biolegend.com/it-it/search-results/purified-anti-human-cd83-antibody-683). The anti CD74 antibody was validated on human peripheral blood lymphocytes (https://sandbox.biolegend.com/it-it/products/purified-anti-human-cd74-antibody-4091). The anti human PCNA antibody was validated on human breast carcinoma tissue samples (https://www.thermofisher.com/antibody/product/PCNA-Antibody-clone-PC10-Monoclonal/13-3900).

Human research participants

Policy information about studies involving human research participants

Population characteristics	Detailed description of the Religious Orders Study and the Memory and Aging Project (ROS/MAP) can be found in the following publications: PMID: 29865057, PMID: 22471860, PMID: 22471867. Information on the brain donation system of the Massachusetts Alzheimer's Disease Center at MGH can be found here: https://www.madrc.org/brain-autopsy-and-donation-information. Detailed description of the brain donation system of the Sun Health Research Institute in Sun City, Arizona can be found here PMID: 18347928. The description of the brain bank at Columbia University Medical Center (New York Brain Bank) can be found here PMID: 29496134. Brain specimens from the BWH included resected tissue of patients suffering from intractable epilepsy. All donors were consented for the use of their tissue for research purposes.
Recruitment	Detailed description of the Religious Orders Study and the Memory and Aging Project (ROS/MAP) can be found in the following publications: PMID: 29865057, PMID: 22471860, PMID: 22471867. Information on the brain donation system of the Massachusetts Alzheimer's Disease Center at MGH can be found here: https://www.madrc.org/brain-autopsy-and-donation-information. Detailed description of the brain donation system of the Sun Health Research Institute in Sun City, Arizona can be found here PMID: 18347928. The description of the brain bank at Columbia University Medical Center (New York Brain Bank) can be found here PMID: 29496134. Brain specimens from the BWH included resected tissue of patients suffering from intractable epilepsy. All donors were consented for the use of their tissue for research purposes.
Ethics oversight	The study has been approved by the ethical committee of: 1) RUSH University, Chicago, IL; 2) Columbia University Medical Center, New York, NY; 3) Brigham and Women's Hospital, Boston, MA.

Note that full information on the approval of the study protocol must also be provided in the manuscript.

Flow Cytometry

Plots

Confirm that:

- [x] The axis labels state the marker and fluorochrome used (e.g. CD4-FITC).
- [x] The axis scales are clearly visible. Include numbers along axes only for bottom left plot of group (a 'group' is an analysis of identical markers).
- [x] All plots are contour plots with outliers or pseudocolor plots.
- [x] A numerical value for number of cells or percentage (with statistics) is provided.

Methodology

Sample preparation	Detailed description of the Religious Orders Study and the Memory and Aging Project (ROS/MAP) can be found in the following publications: PMID: 29865057, PMID: 22471860, PMID: 22471867. Information on the brain donation system of the Massachusetts Alzheimer's Disease Center at MGH can be found here: https://www.madrc.org/brain-autopsy-and-donation-information. Detailed description of the brain donation system of the Sun Health Research Institute in Sun City, Arizona can be found here PMID: 18347928. The description of the brain bank at Columbia University Medical Center (New York Brain Bank) can be found here PMID: 29496134. Brain specimens from the BWH included resected tissue of patients suffering from intractable epilepsy. All donors were consented for the use of their tissue for research purposes.
Instrument	BD's Aria IIu and BD Influx sorters were used for fluorescent activated cell sorting of microglia cells from human brain.
Software	BD's FACSDiva version 8.0.1. software was used during fluorescent activated cell sorting of microglia cells from human brain.
Cell population abundance	Microglia cells represented on average 0.4% of all the events. Among the 7AAD- live cells the CD11b+/CD45+ cells represented ~50% (please note that RBC will also show up as 7AAD-, since they lack a nucleus). The analysis of the sorted cells showed that they were ~99% microglia (CD11b+/CD45+/7AAD-) cells.
Gating strategy	The detailed description of the gating strategy was included in our previous publication (https://www.nature.com/articles/s41467-018-02926-5; PMID: 29416036). Briefly, cells were gated on the FSC/SSC scatter plots (Gate 1), from which the dead cells were excluded based on their 7AAD positivity (Gate 2: 7AAD- events). The third gate was placed on the CD11b/CD45 double positive events (Gate 3: CD11b+/CD45+).

- [x] Tick this box to confirm that a figure exemplifying the gating strategy is provided in the Supplementary Information.
